# Supplementary material for: Human DNA polymerase delta is a pentameric holoenzyme with a dimeric p12 subunit
Source: Life Sci Alliance. 2019 Mar 18;2(2):e201900323. doi: 10.26508/lsa.201900323 (PMC6424025; doi:10.26508/lsa.201900323)

Raw data for Figure 1A .

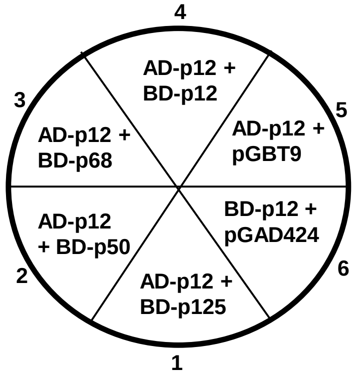

Leu$^-$ Trp$^-$

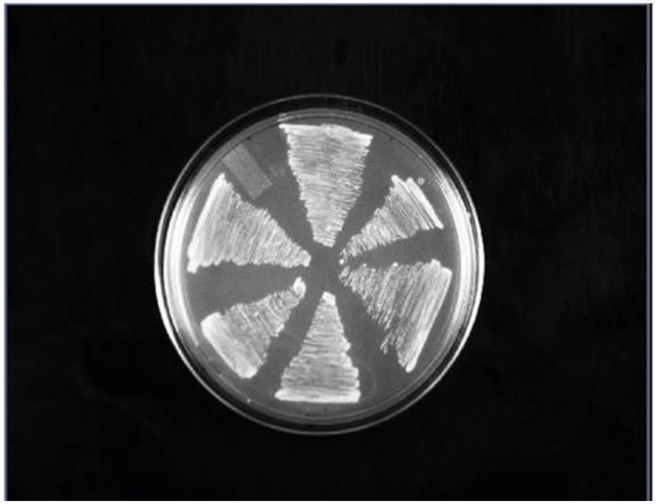

Leu$^-$ Trp$^-$ His$^-$

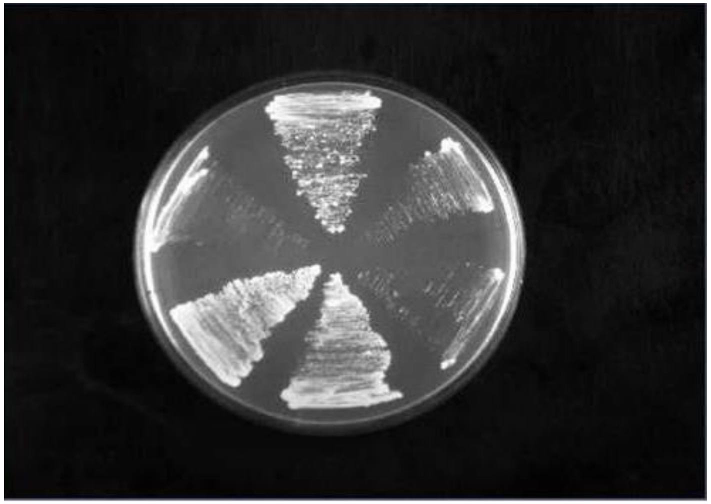

Raw data for Figure 1B .

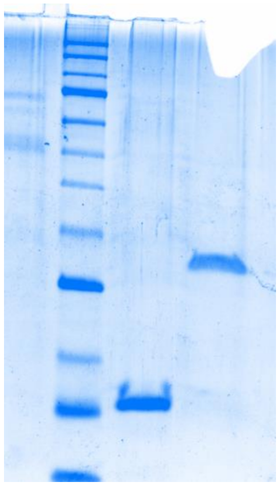

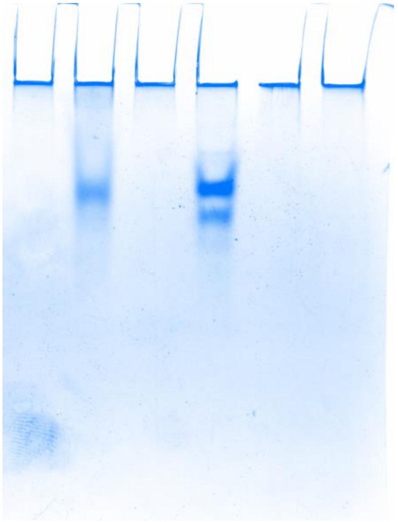

P12 sds gel

P12 native gel

Raw plot for F igure 1C i (p12 –p12)

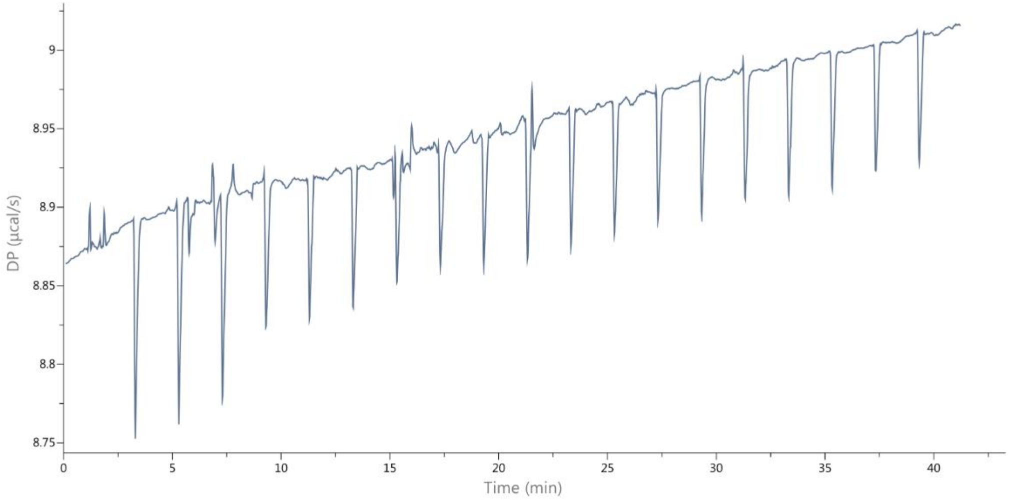

Raw plot for figure 1C ii (p12- RKR)

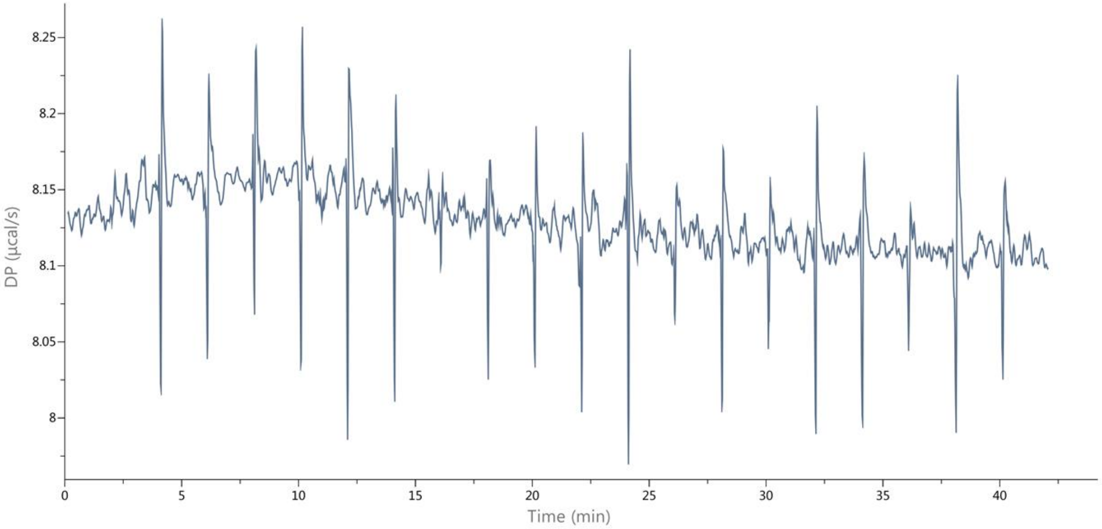

Supplement: Supplementary file 1 [file LSA-2019-00323_SdataF1.pdf]
