# Supplementary material for: Human DNA polymerase delta is a pentameric holoenzyme with a dimeric p12 subunit
Source: Life Sci Alliance. 2019 Mar 18;2(2):e201900323. doi: 10.26508/lsa.201900323 (PMC6424025; doi:10.26508/lsa.201900323)

Raw figures for Figure 2A.

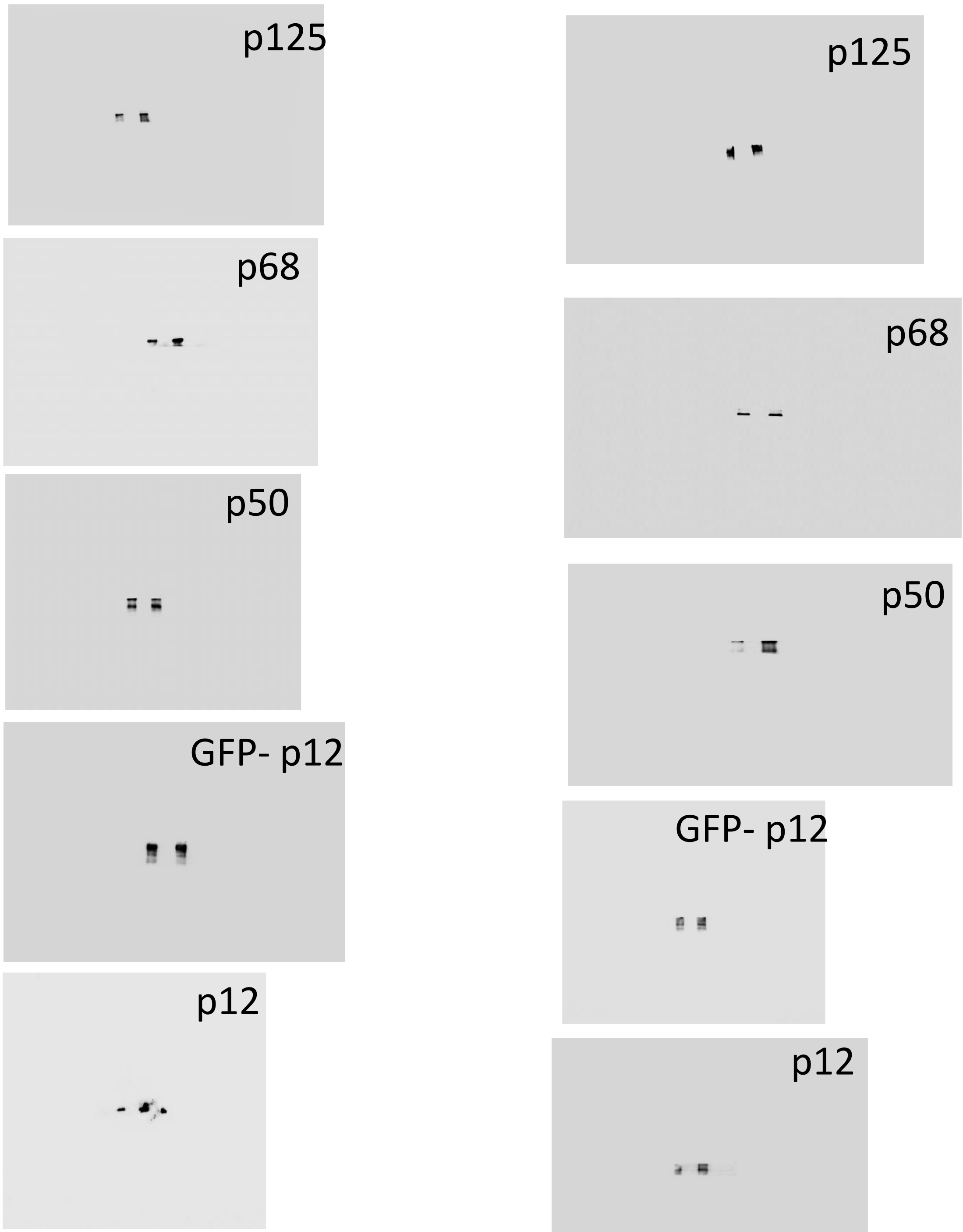

Raw data for Figure 2B i (GFP-p12 and RFP-p12) .

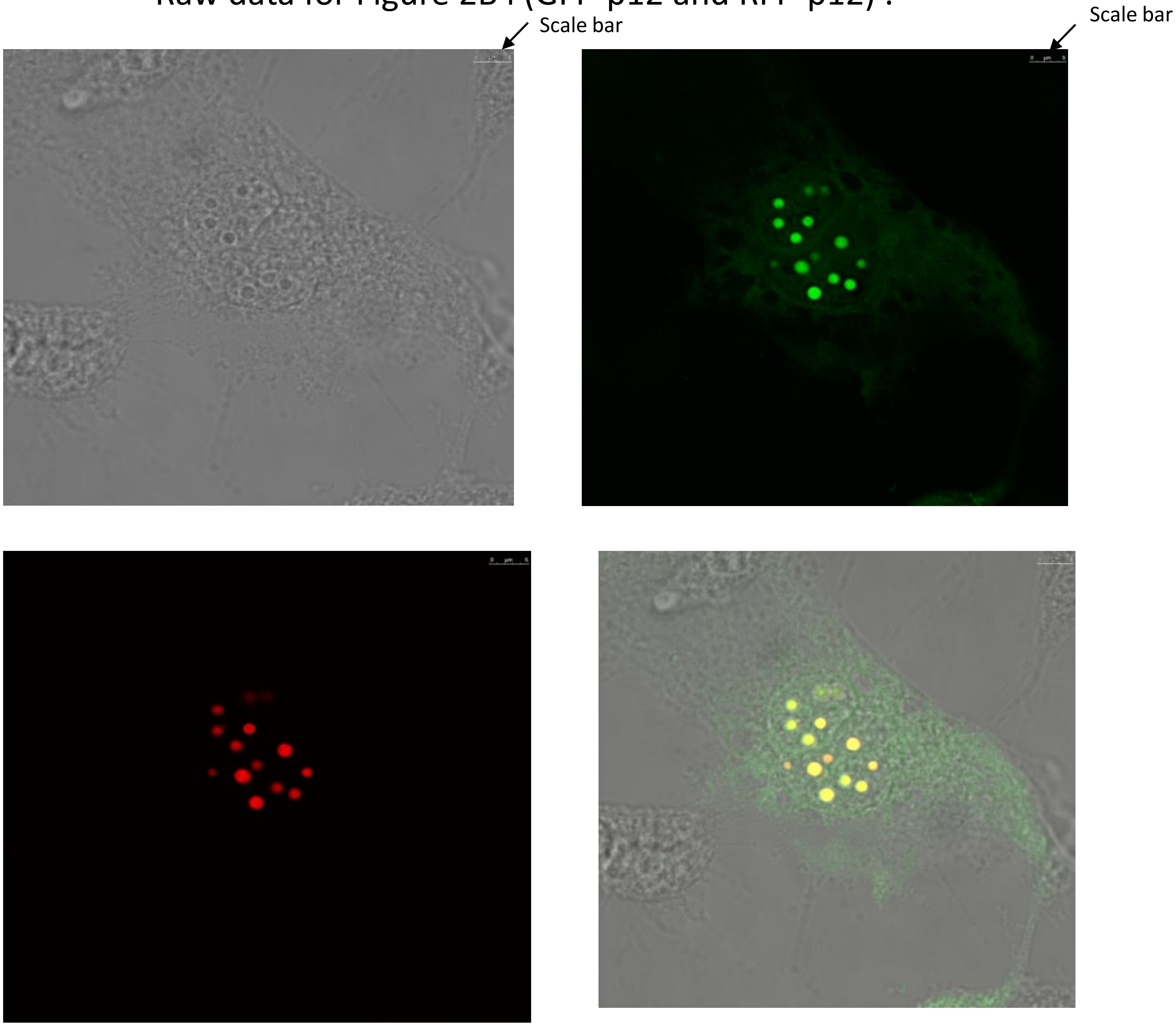

Raw data for Figure 2B ii (GFP-PCNA and RFP-p12) .

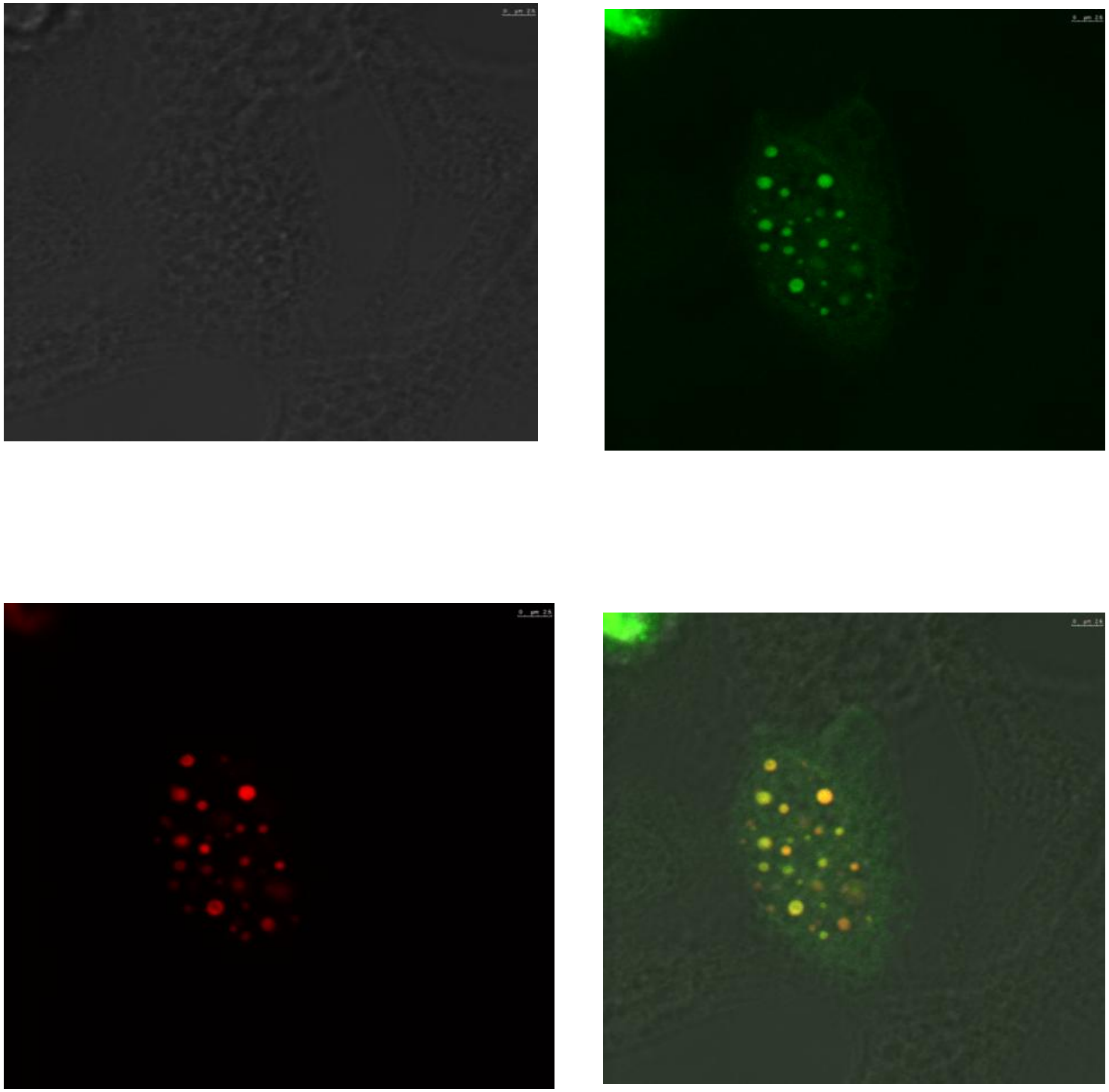

Raw data for figure 2B iii (GFP-p12 and RFP-Polθ) .

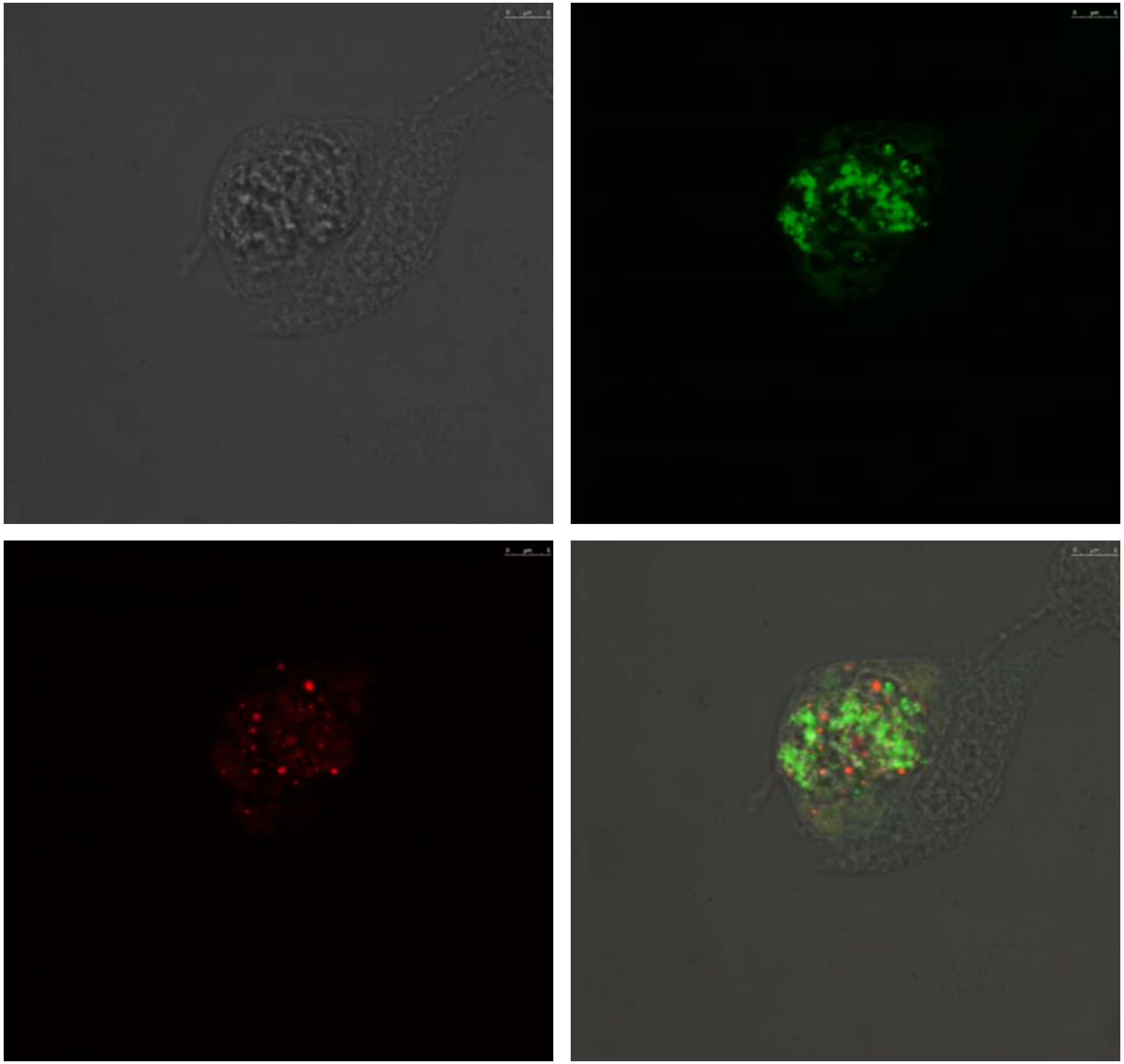

Supplement: Supplementary file 2 [file LSA-2019-00323_SdataF2.pdf]
