# Supplementary figures and images for: Human DNA polymerase delta is a pentameric holoenzyme with a dimeric p12 subunit
Source: Life Sci Alliance. 2019 Mar 18;2(2):e201900323. doi: 10.26508/lsa.201900323 (PMC6424025; doi:10.26508/lsa.201900323)

Raw data for Figure 3D .

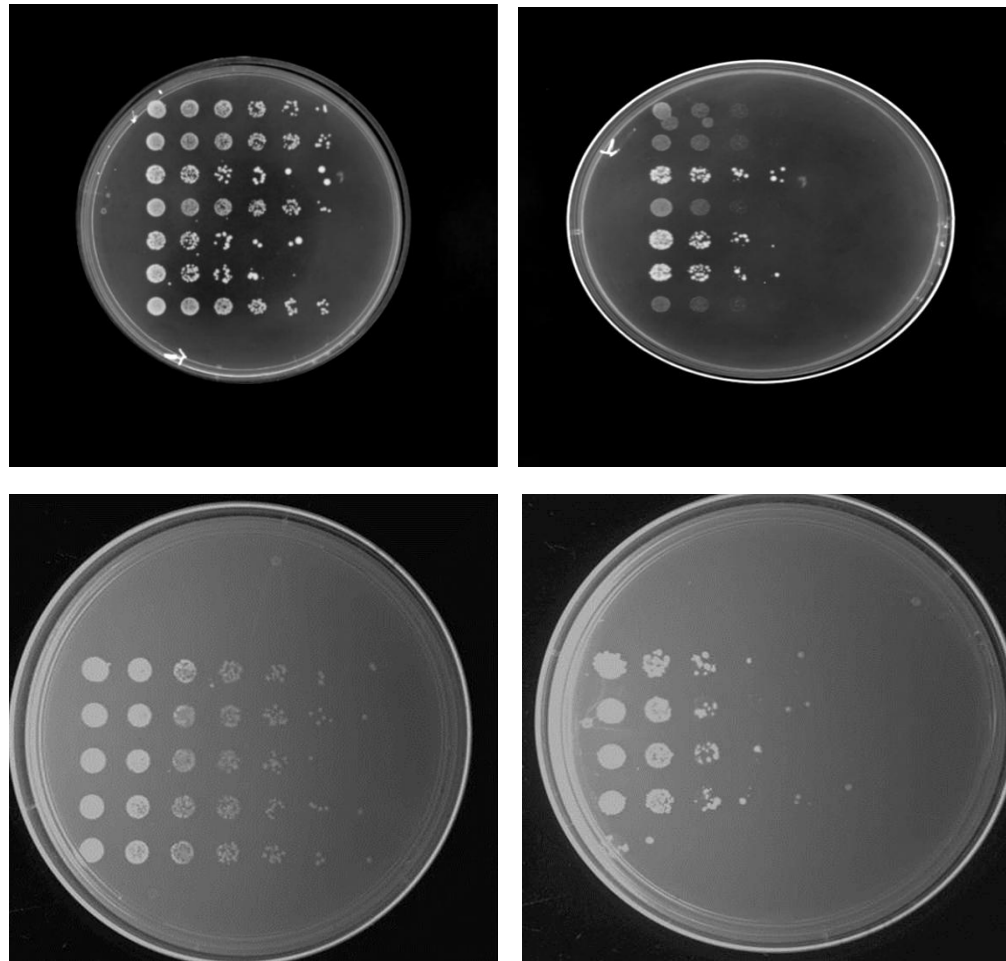

Supplement: Supplementary file 3 [file LSA-2019-00323_SdataF3.pdf]

4A.

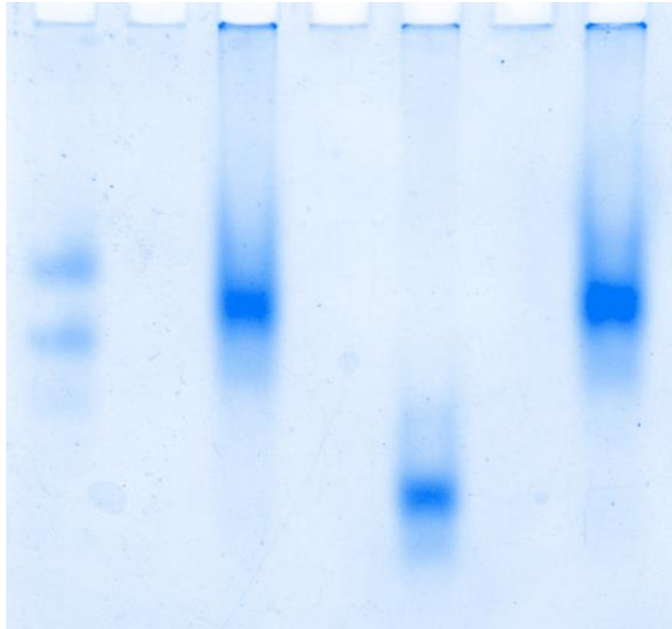

4 B.

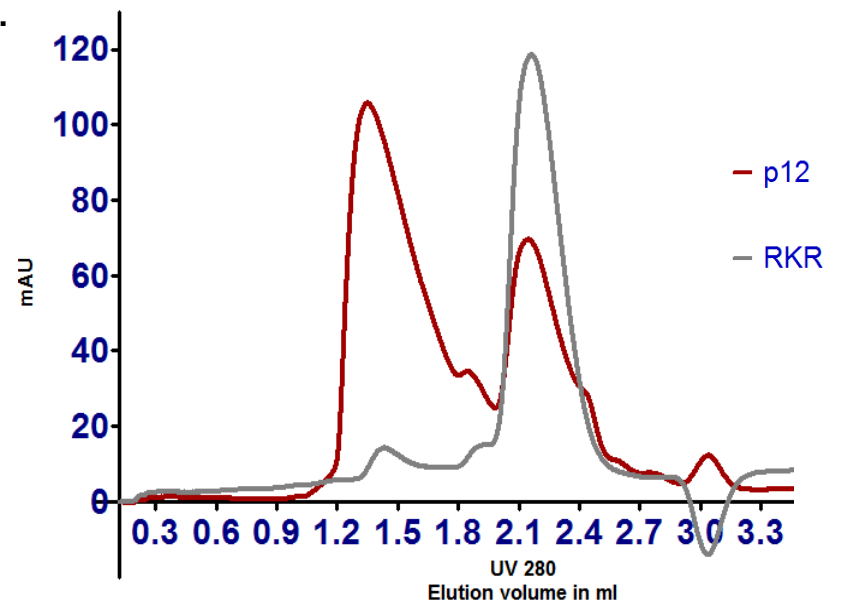

Raw data for figure 4C .

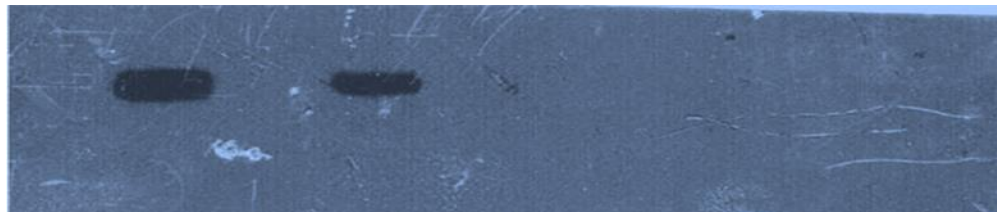

Supplement: Supplementary file 4 [file LSA-2019-00323_SdataF4.pdf]

Raw Figure 5 A

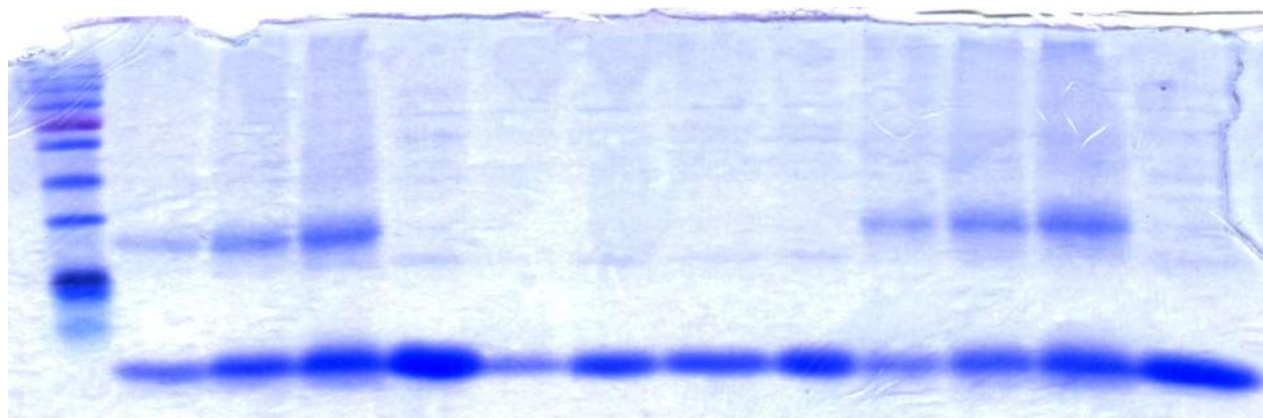

Supplement: Supplementary file 5 [file LSA-2019-00323_SdataF5A.pdf]

Chemidoc-Raw figure for Figure 6A.

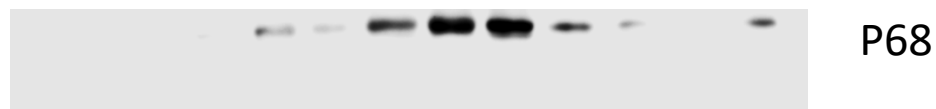

Chemidoc-Raw figure for Figure 6B.

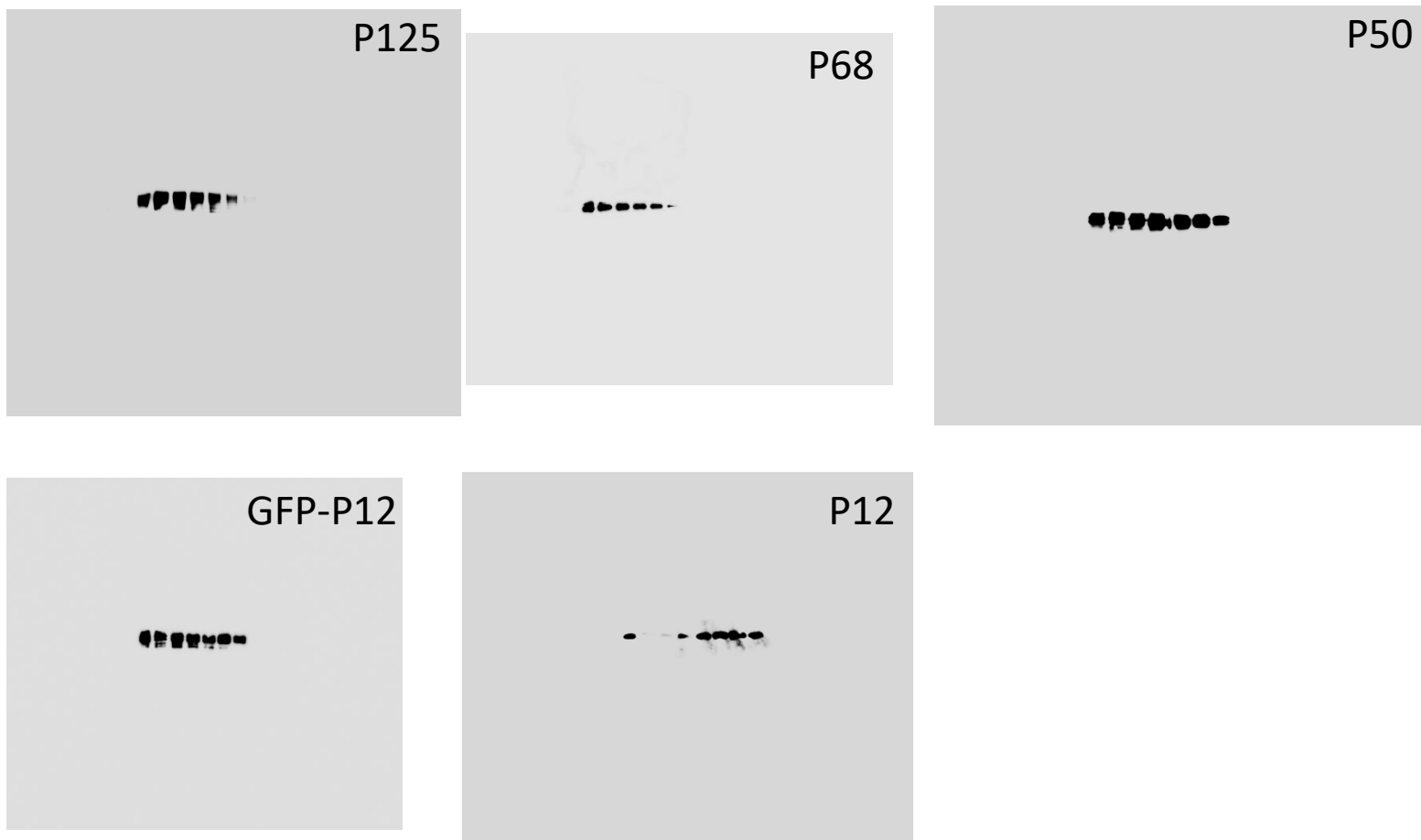

Supplement: Supplementary file 7 [file LSA-2019-00323_SdataF6.pdf]

Raw figure for Figure 8A

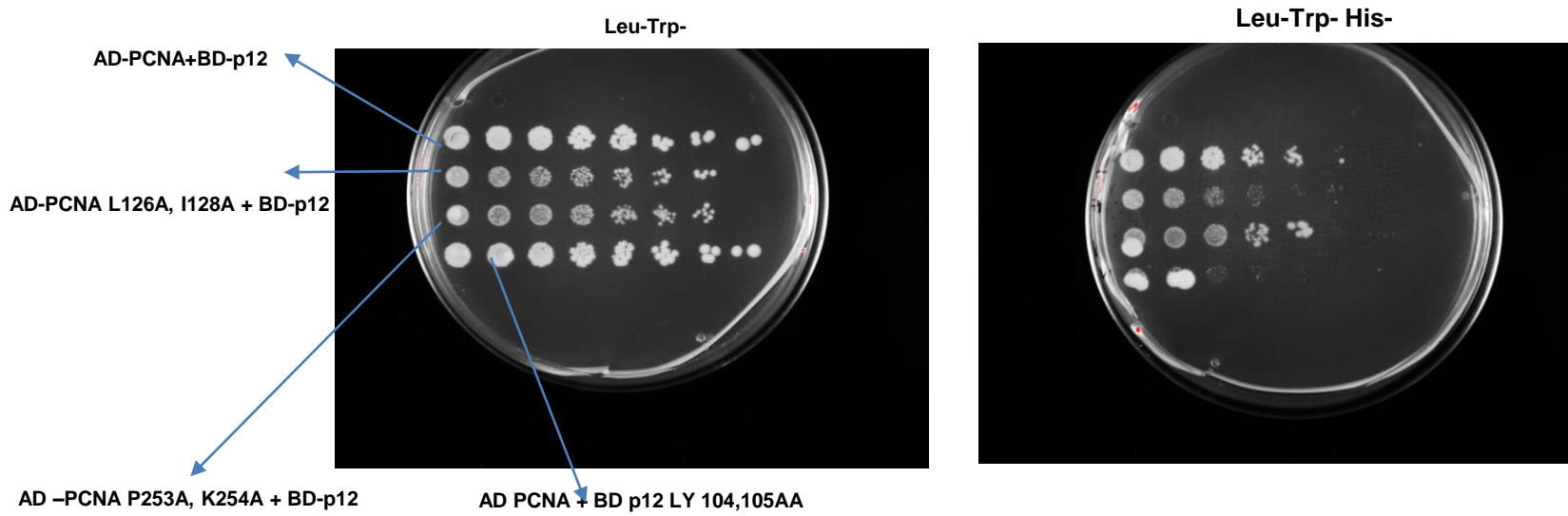

Chemidoc- Raw figure for Figure 8B.

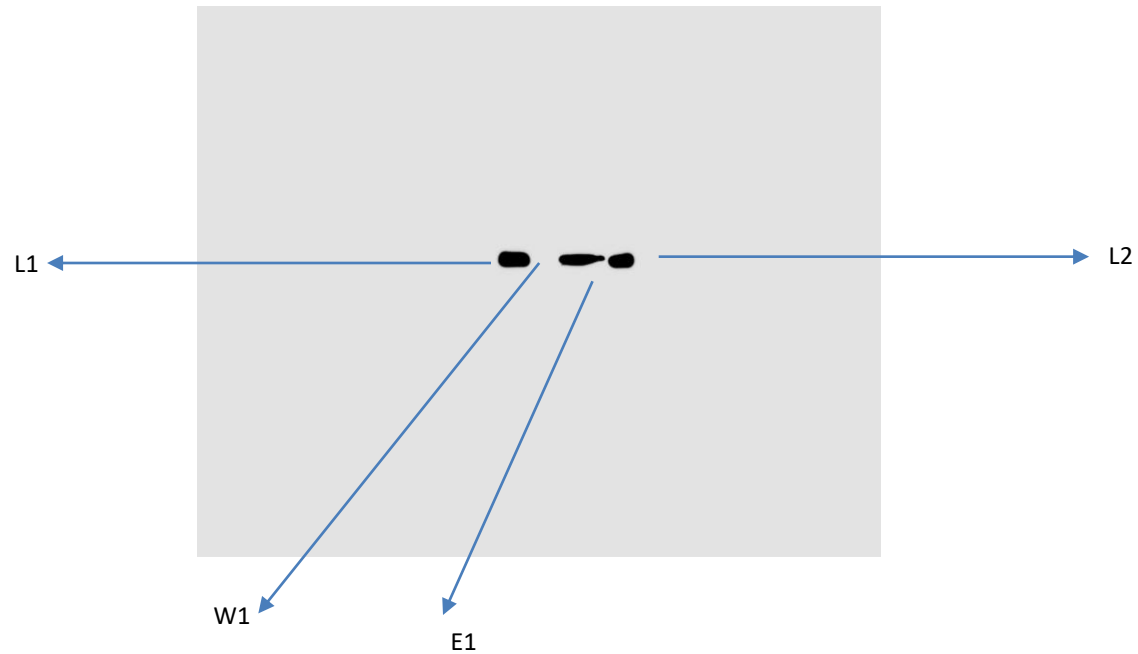

Supplement: Supplementary file 9 [file LSA-2019-00323_SdataF8.pdf]

Raw figure for Figure 9

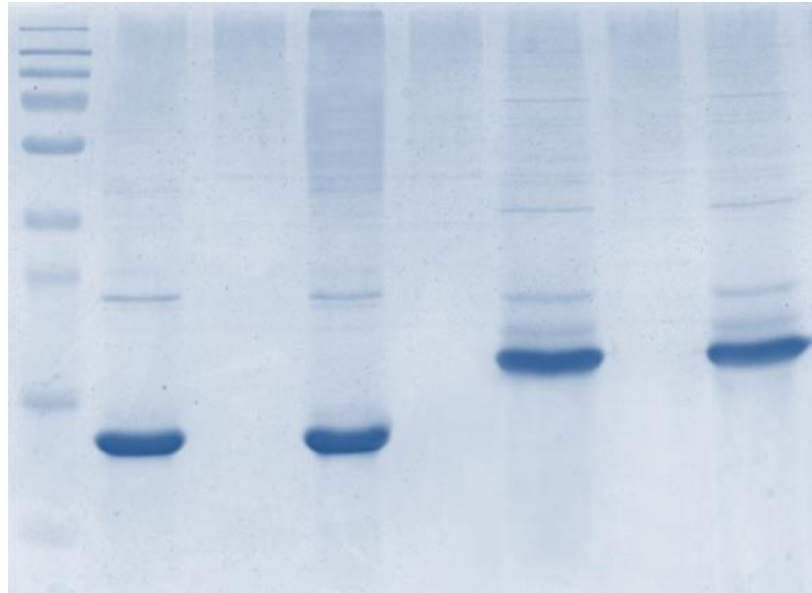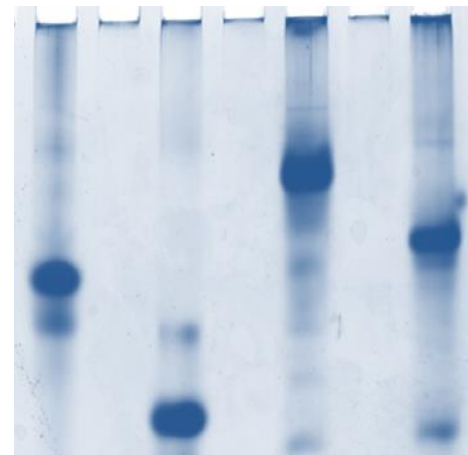

Supplement: Supplementary file 10 [file LSA-2019-00323_SdataF9.pdf]

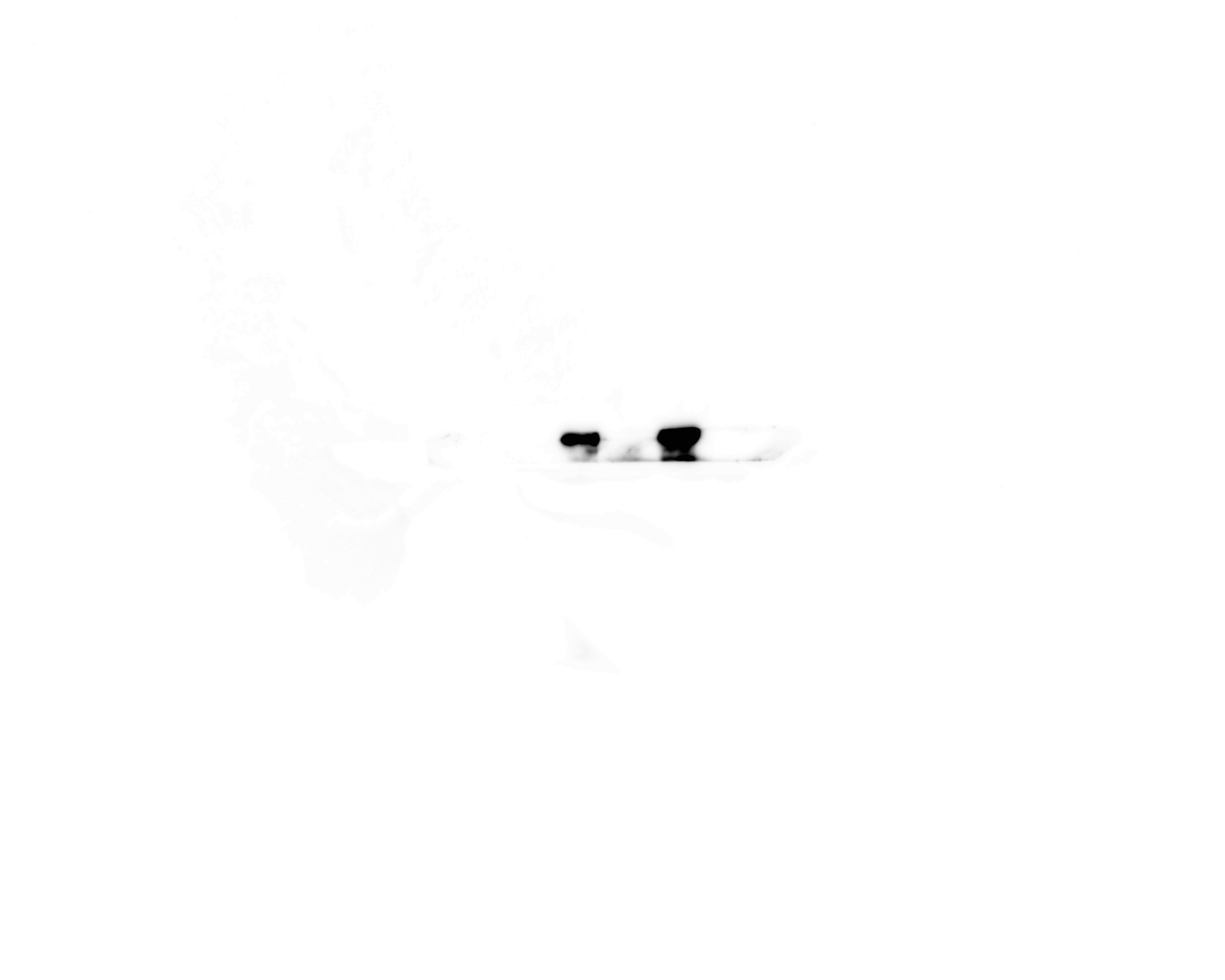

Supplement: Supplementary file 11 [file LSA-2019-00323_SdataF10.zip › Acharya_westernBlot_SourceData/2Ai_prashant 2018-12-04 04h29m49s p68 ip pull by gfp p12 panel1.tif]

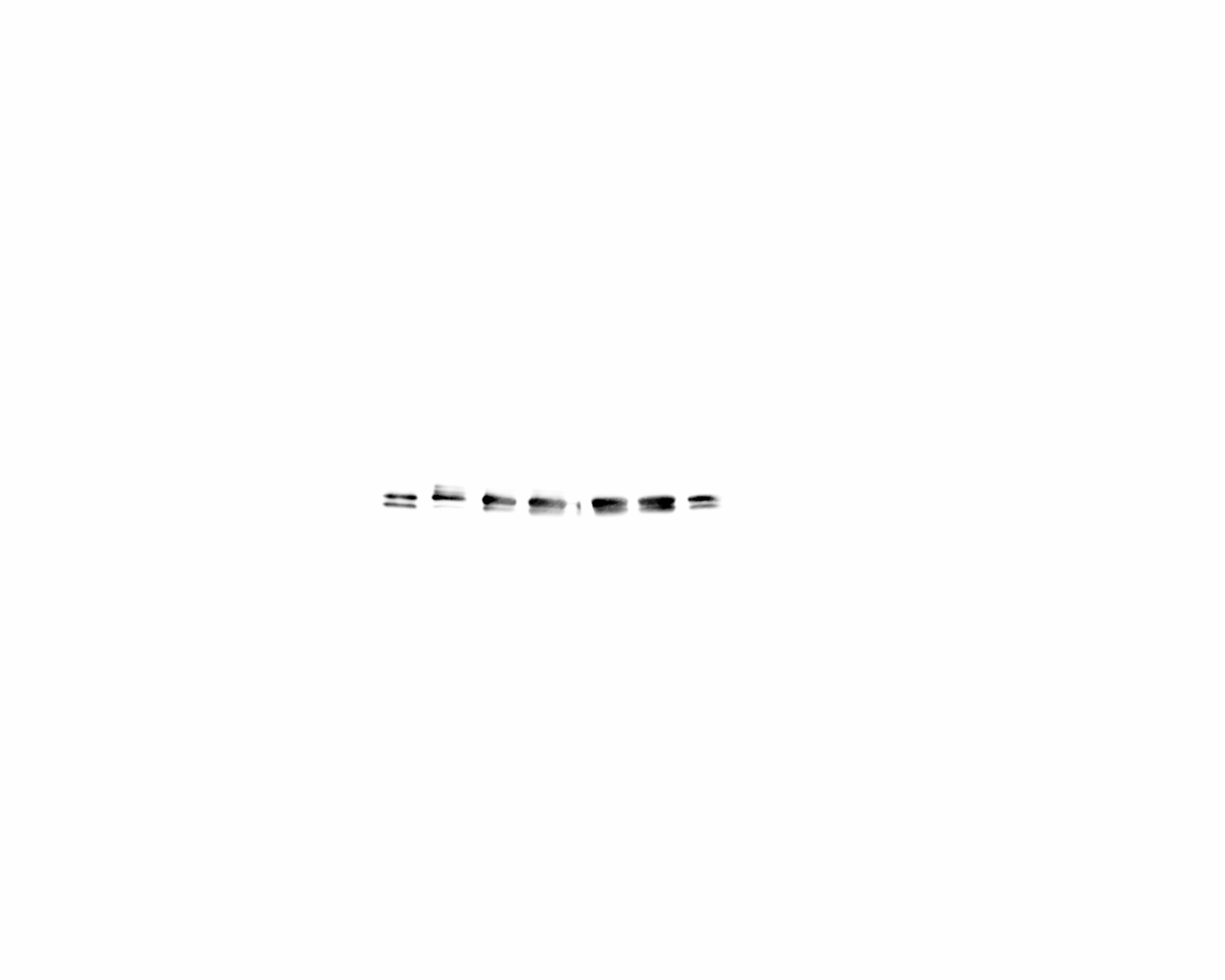

Supplement: Supplementary file 11 [file LSA-2019-00323_SdataF10.zip › Acharya_westernBlot_SourceData/6B_prashant 2018-12-26 07h11m43s anti p50 after fplc.tif]

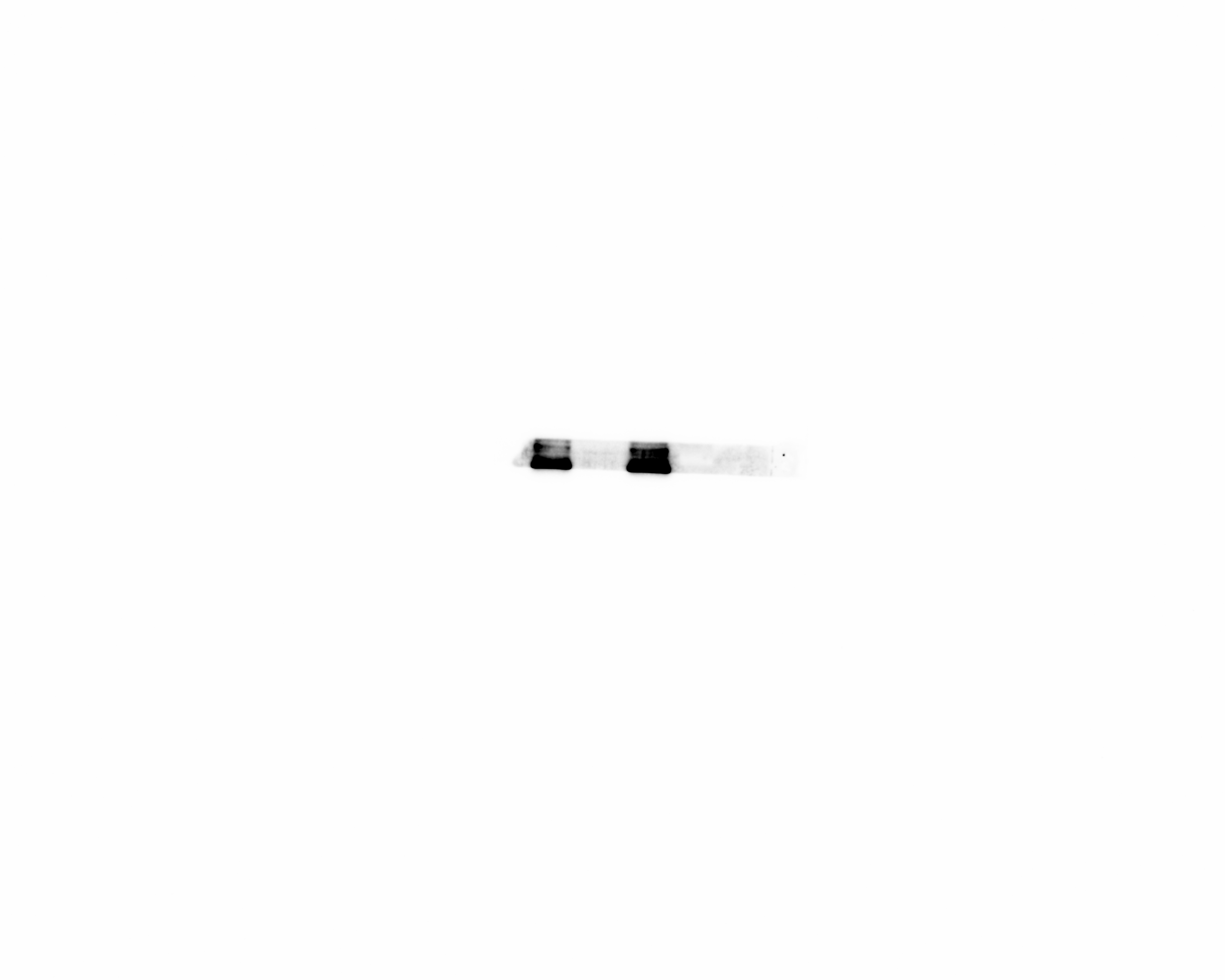

Supplement: Supplementary file 11 [file LSA-2019-00323_SdataF10.zip › Acharya_westernBlot_SourceData/2Aii_prashant 2018-12-12 04h15m25s p68 ip panel2.tif]

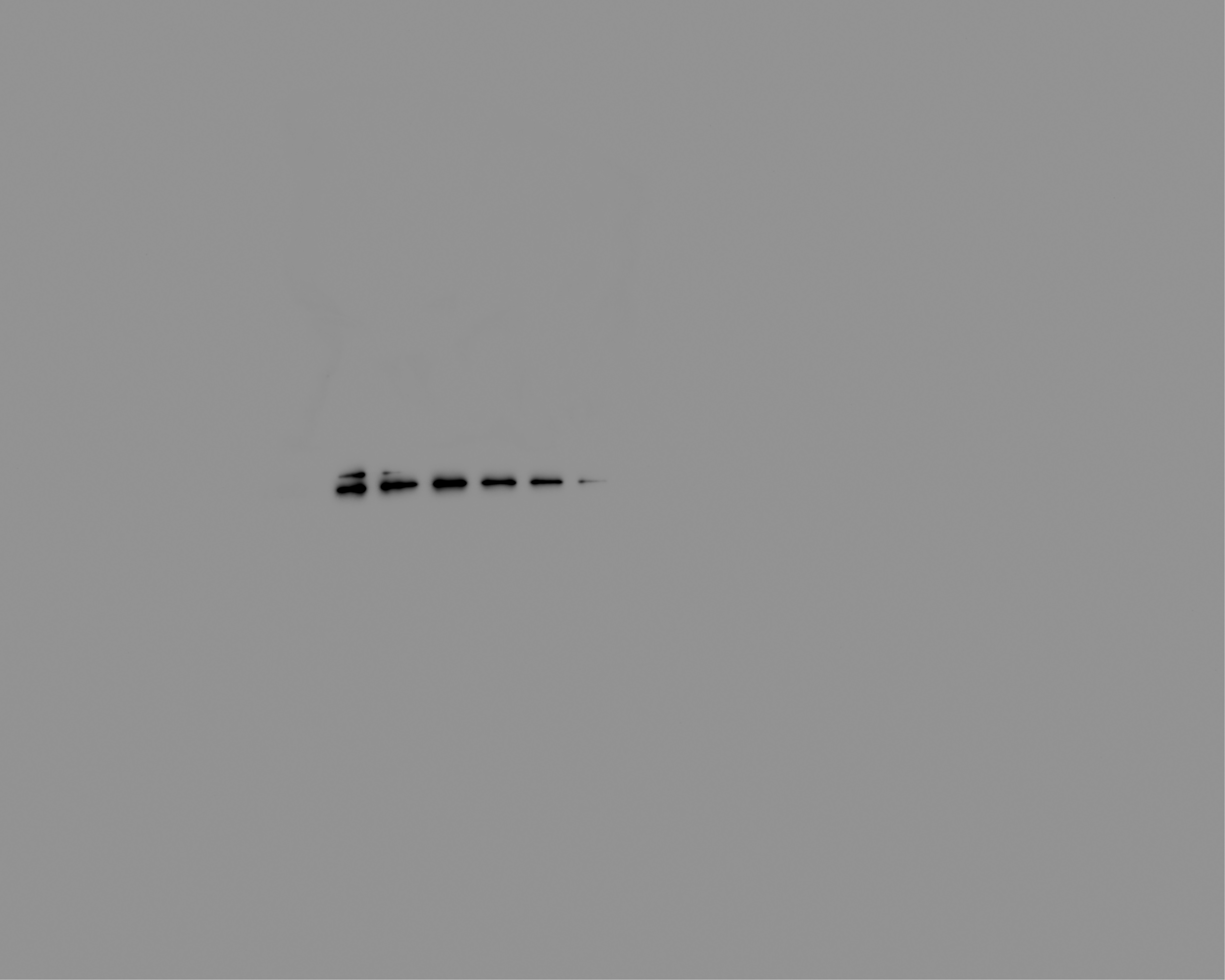

Supplement: Supplementary file 11 [file LSA-2019-00323_SdataF10.zip › Acharya_westernBlot_SourceData/6B_prashant 2018-12-26 03h21m54s p68 fplc.tif]

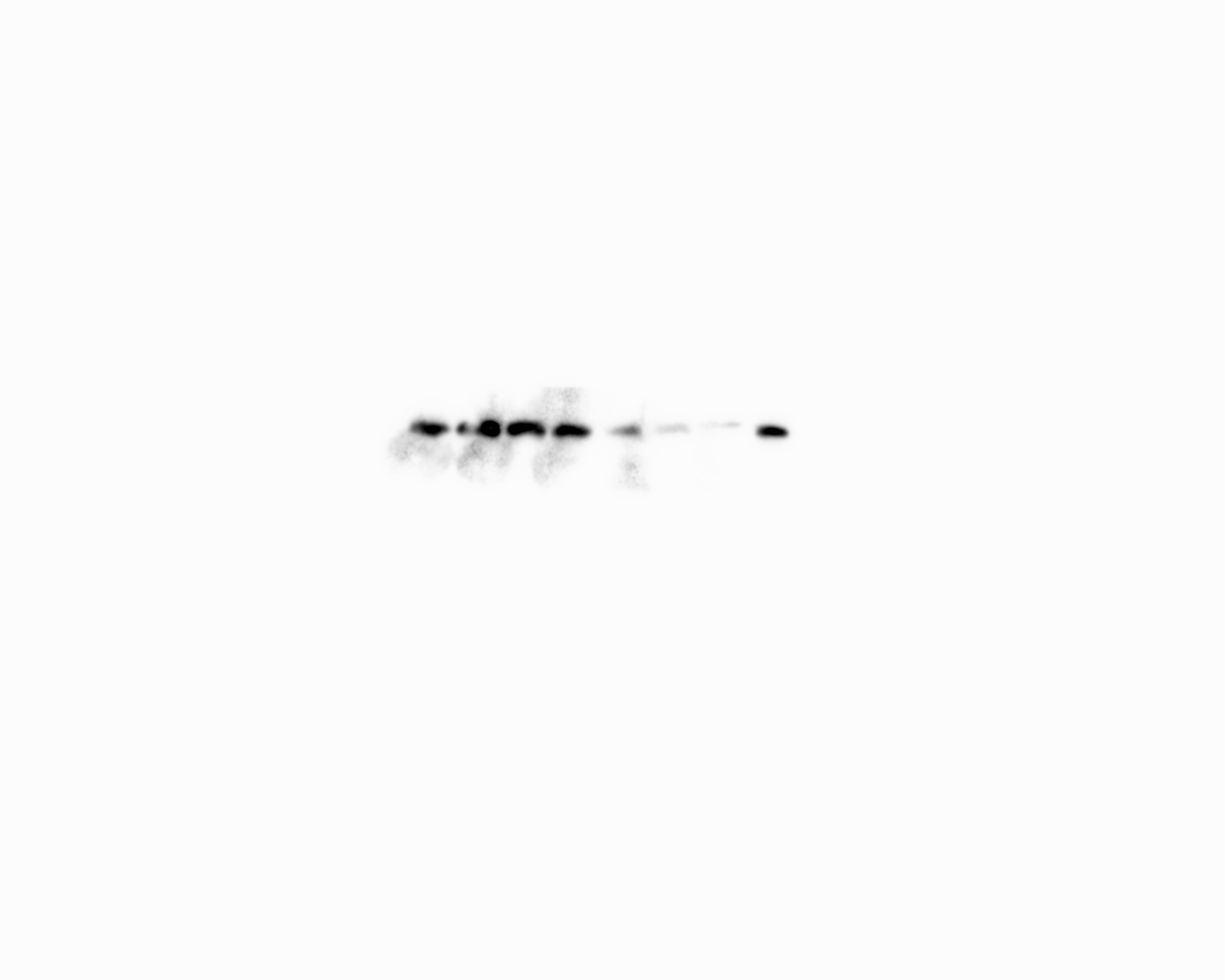

Supplement: Supplementary file 11 [file LSA-2019-00323_SdataF10.zip › Acharya_westernBlot_SourceData/6B_prashant 2018-12-26 03h39m59s p12 at lower position.tif]

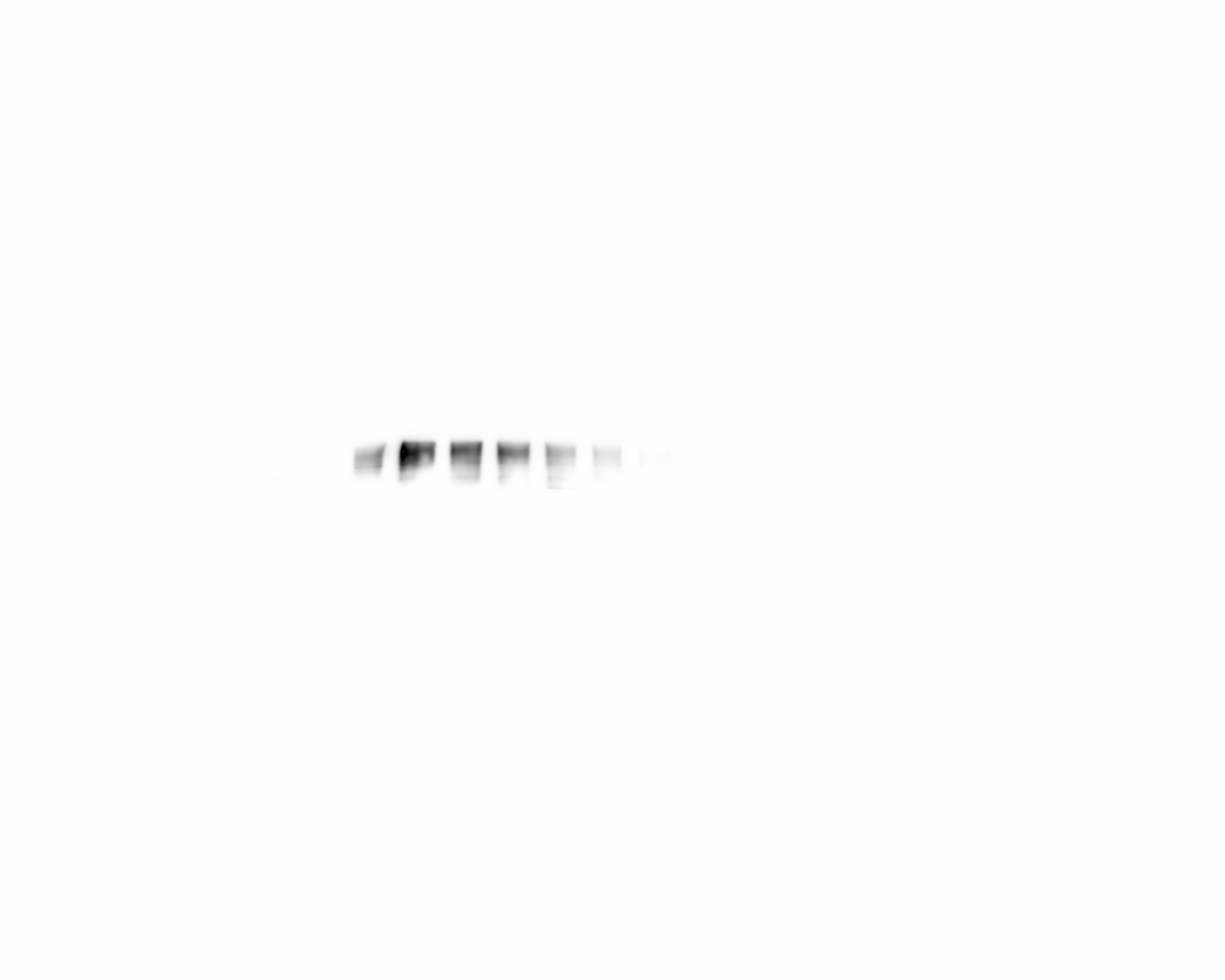

Supplement: Supplementary file 11 [file LSA-2019-00323_SdataF10.zip › Acharya_westernBlot_SourceData/6Bprashant 2018-12-26 03h27m33sp 125.tif]

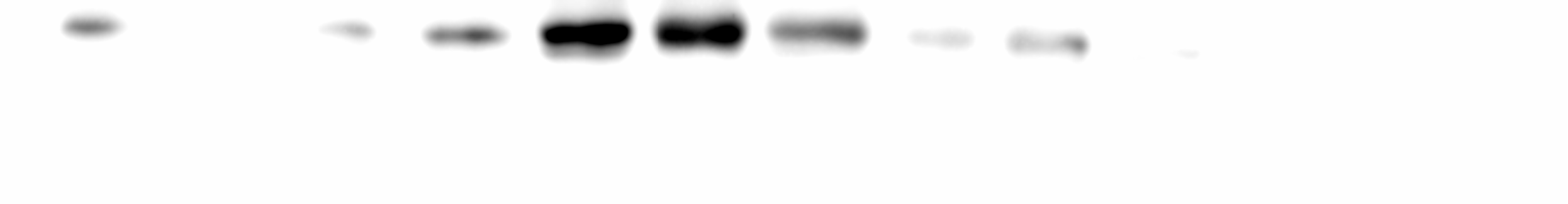

Supplement: Supplementary file 11 [file LSA-2019-00323_SdataF10.zip › Acharya_westernBlot_SourceData/6A_prashant 2018-12-24 21h47m04s p68 pol delta after fplc fig.tif]

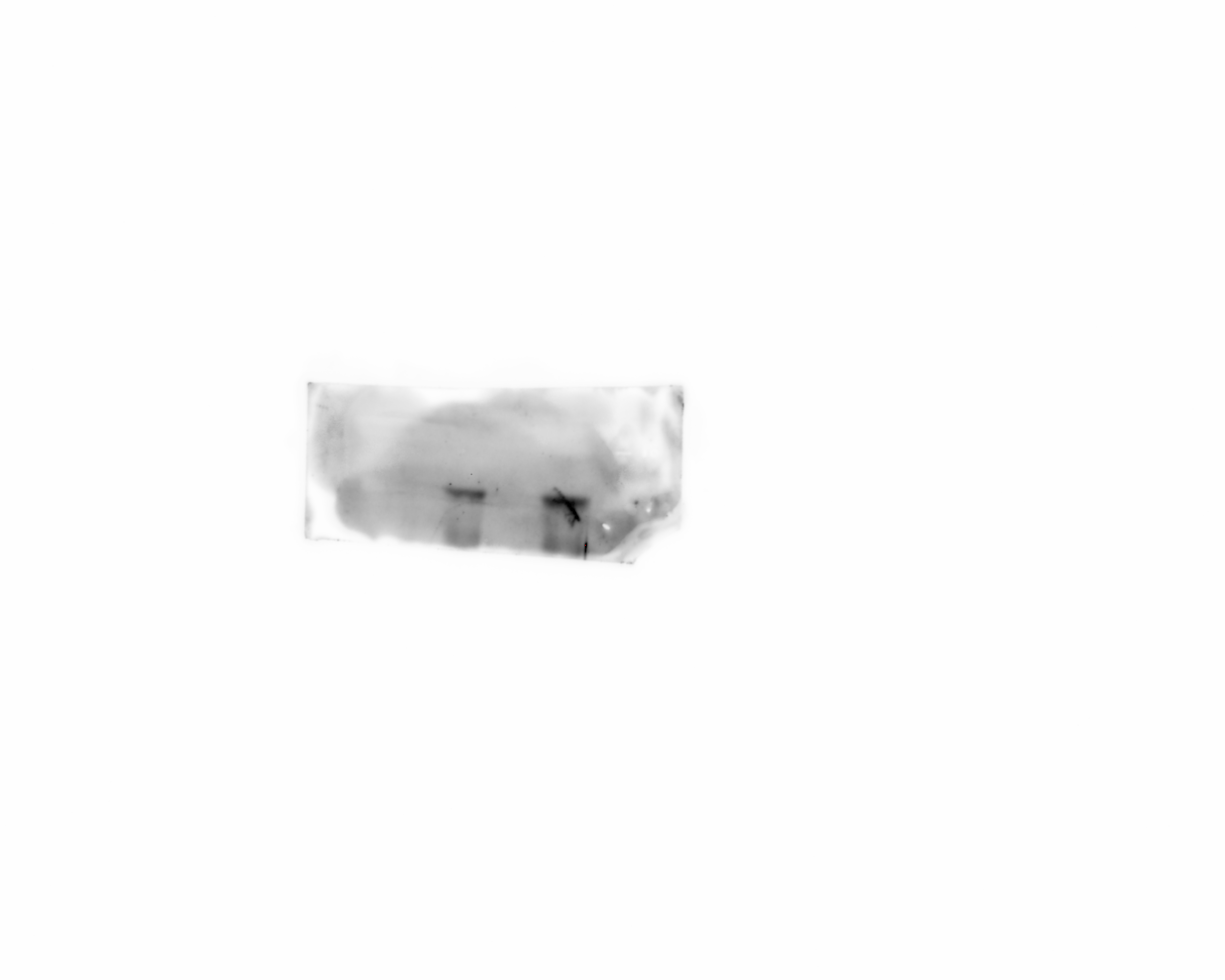

Supplement: Supplementary file 11 [file LSA-2019-00323_SdataF10.zip › Acharya_westernBlot_SourceData/2Ai_prashant 2018-12-06 04h50m53sp12 panel1.tif]

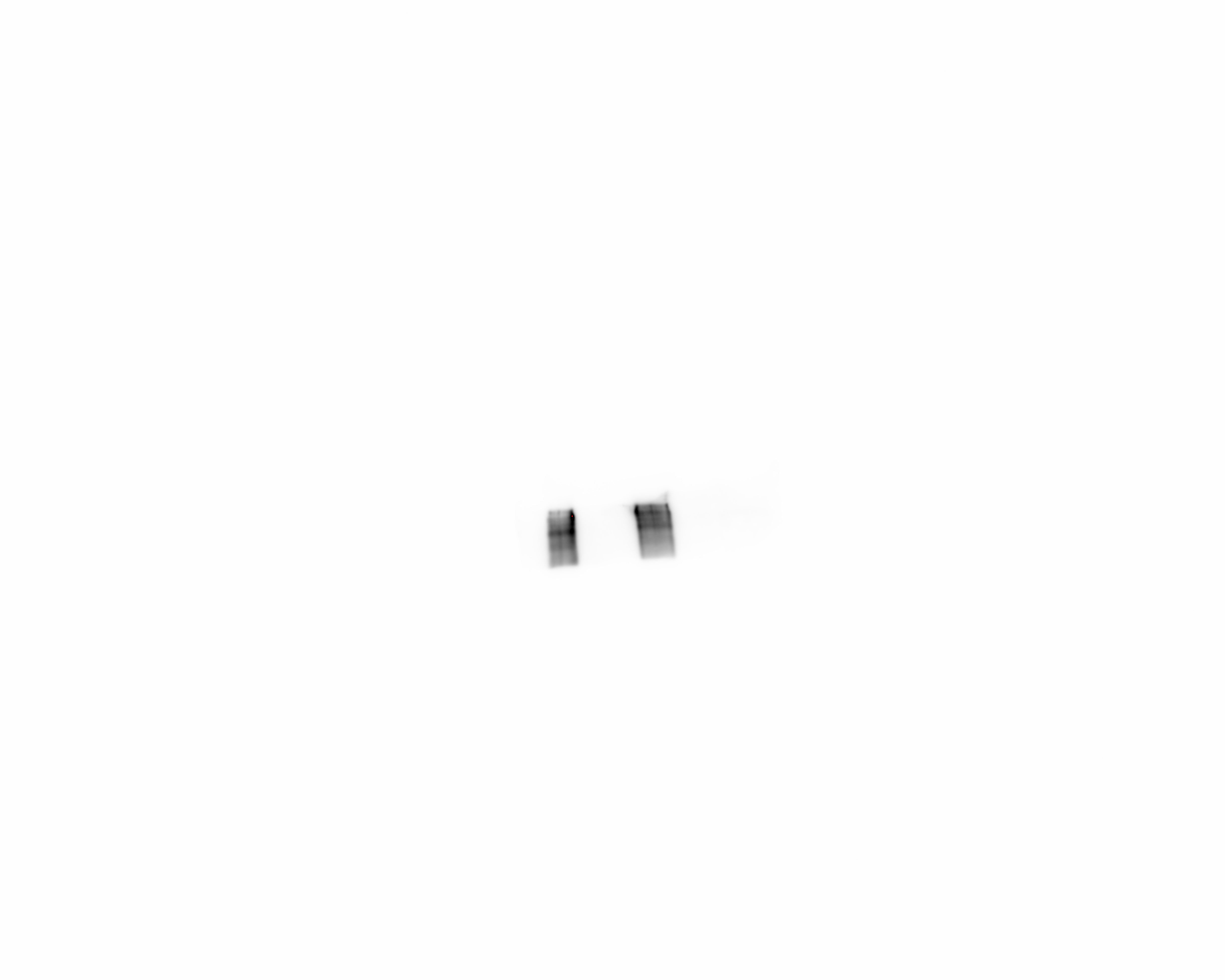

Supplement: Supplementary file 11 [file LSA-2019-00323_SdataF10.zip › Acharya_westernBlot_SourceData/2Aii_prashant 2018-12-15 02h30m17s p125 by ip using anti p125 panel2.tif]

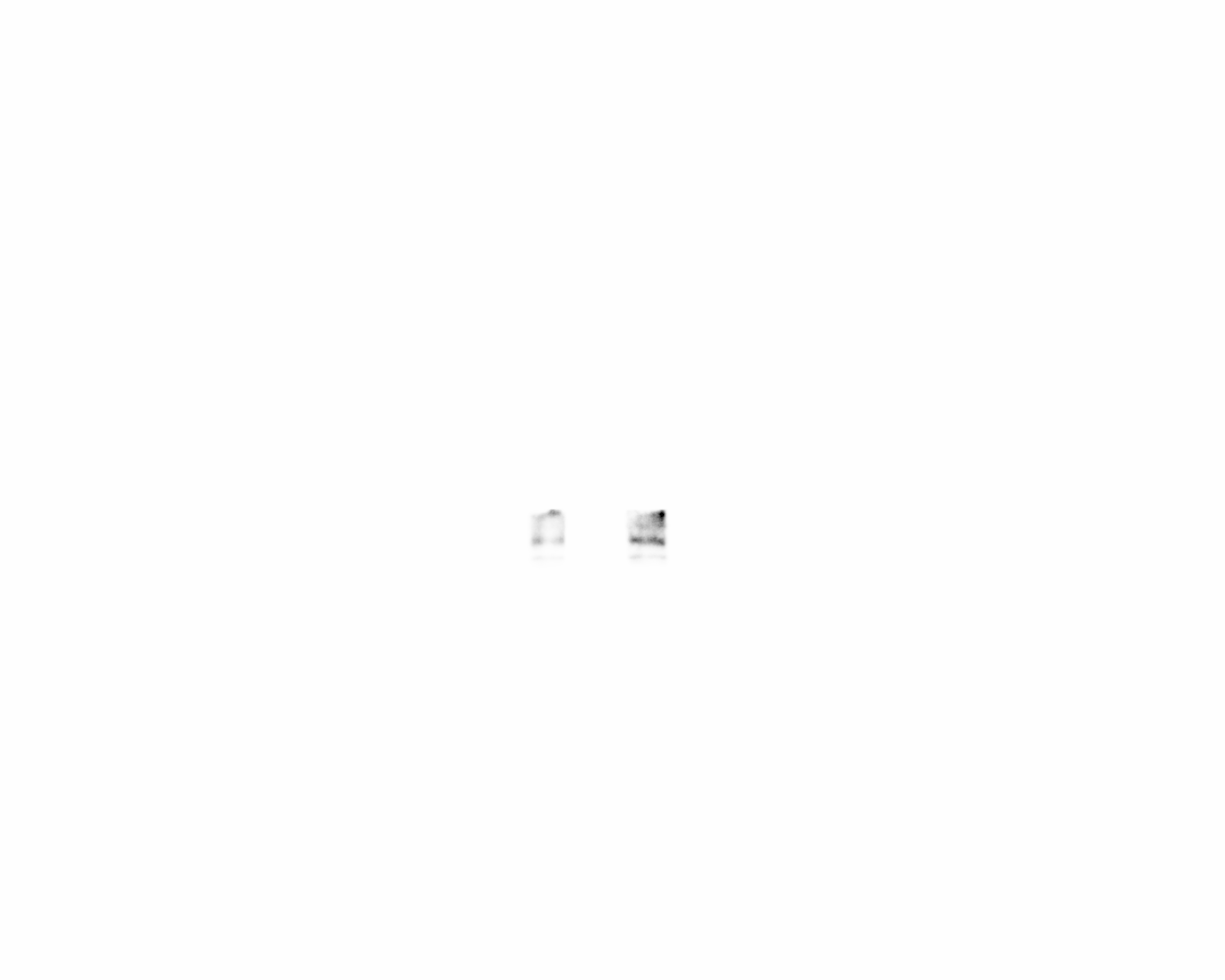

Supplement: Supplementary file 11 [file LSA-2019-00323_SdataF10.zip › Acharya_westernBlot_SourceData/2Aii_prashant-2018-12-06 03h28m30s gfp p12 ip (Panel II).tif]

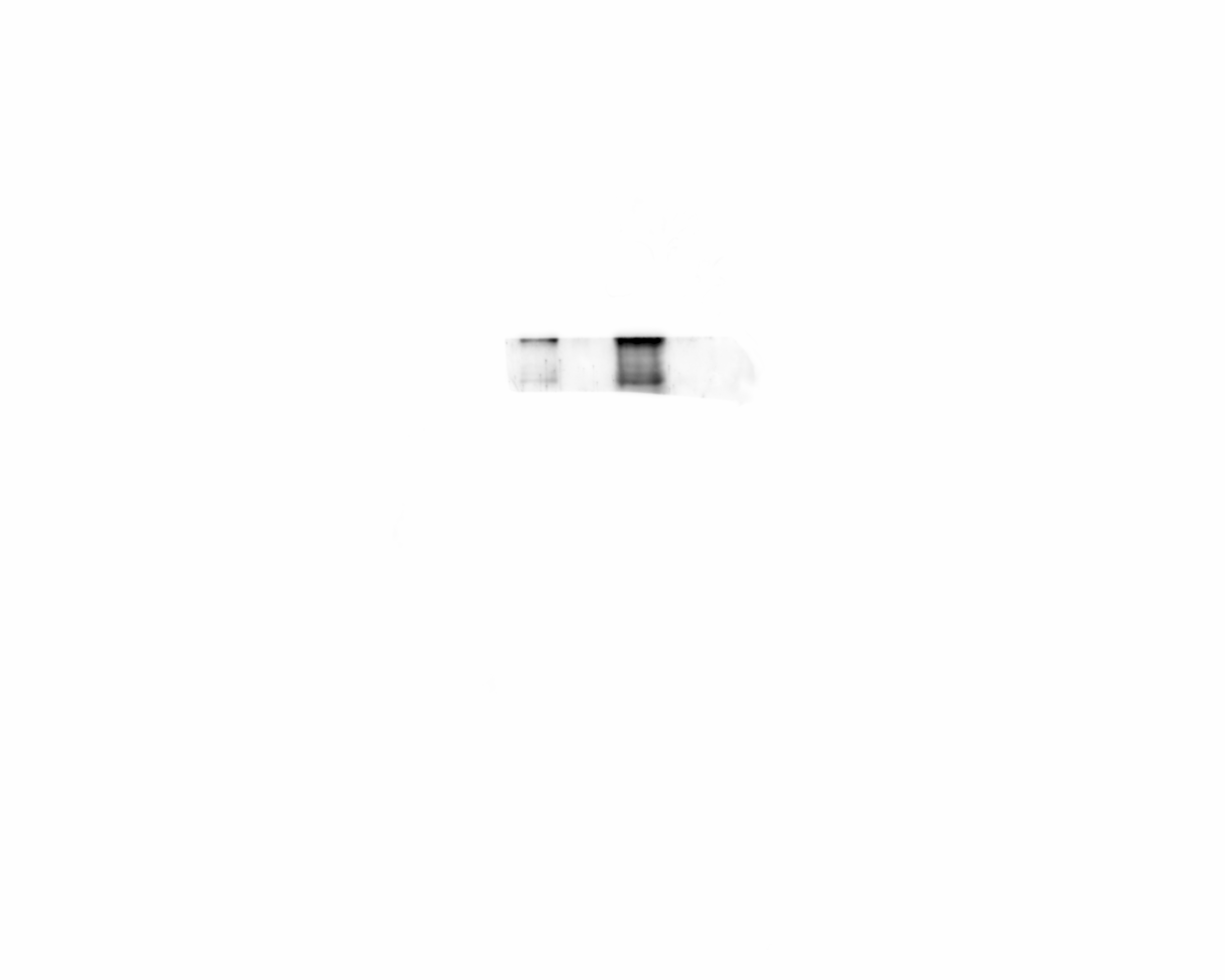

Supplement: Supplementary file 11 [file LSA-2019-00323_SdataF10.zip › Acharya_westernBlot_SourceData/2Aii_prashant 2018-12-12 ipp50 panel2.tif]

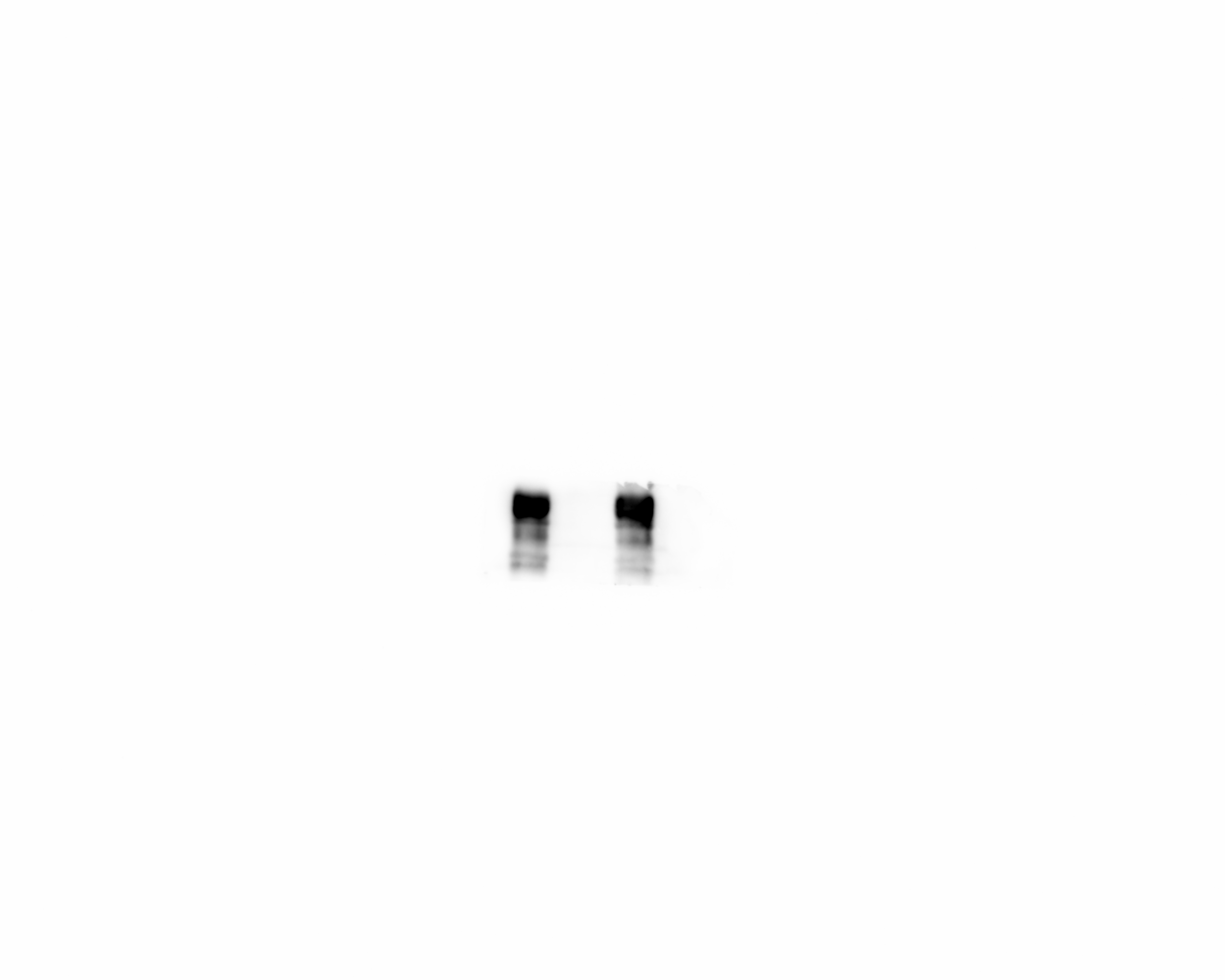

Supplement: Supplementary file 11 [file LSA-2019-00323_SdataF10.zip › Acharya_westernBlot_SourceData/2Ai_prashant 2018-12-15 03h25m19s gfp p12 ip panel1.tif]

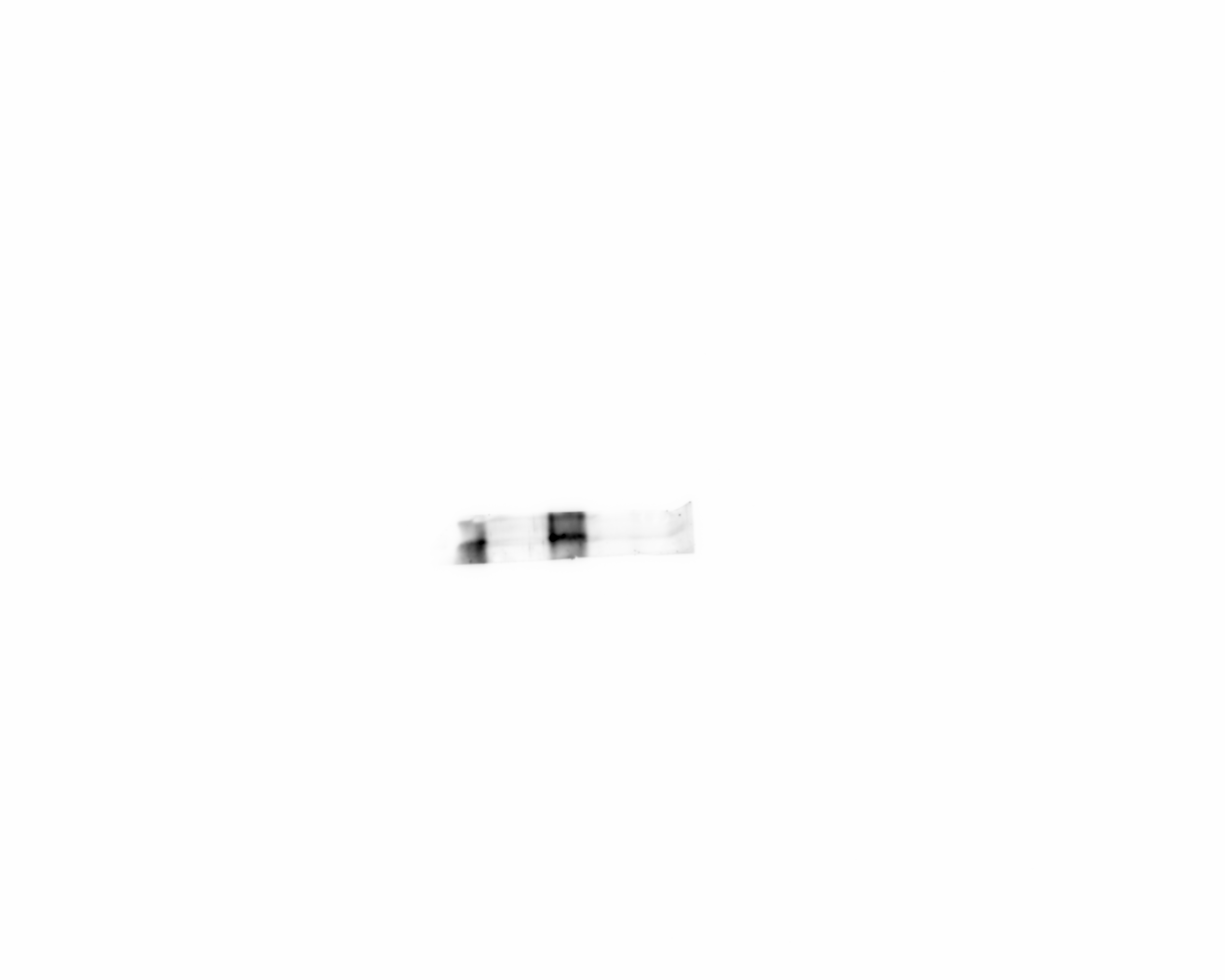

Supplement: Supplementary file 11 [file LSA-2019-00323_SdataF10.zip › Acharya_westernBlot_SourceData/2Aii_prashant 2018-12-15 03h15m49s p12 ip n by p125 panel2.tif]

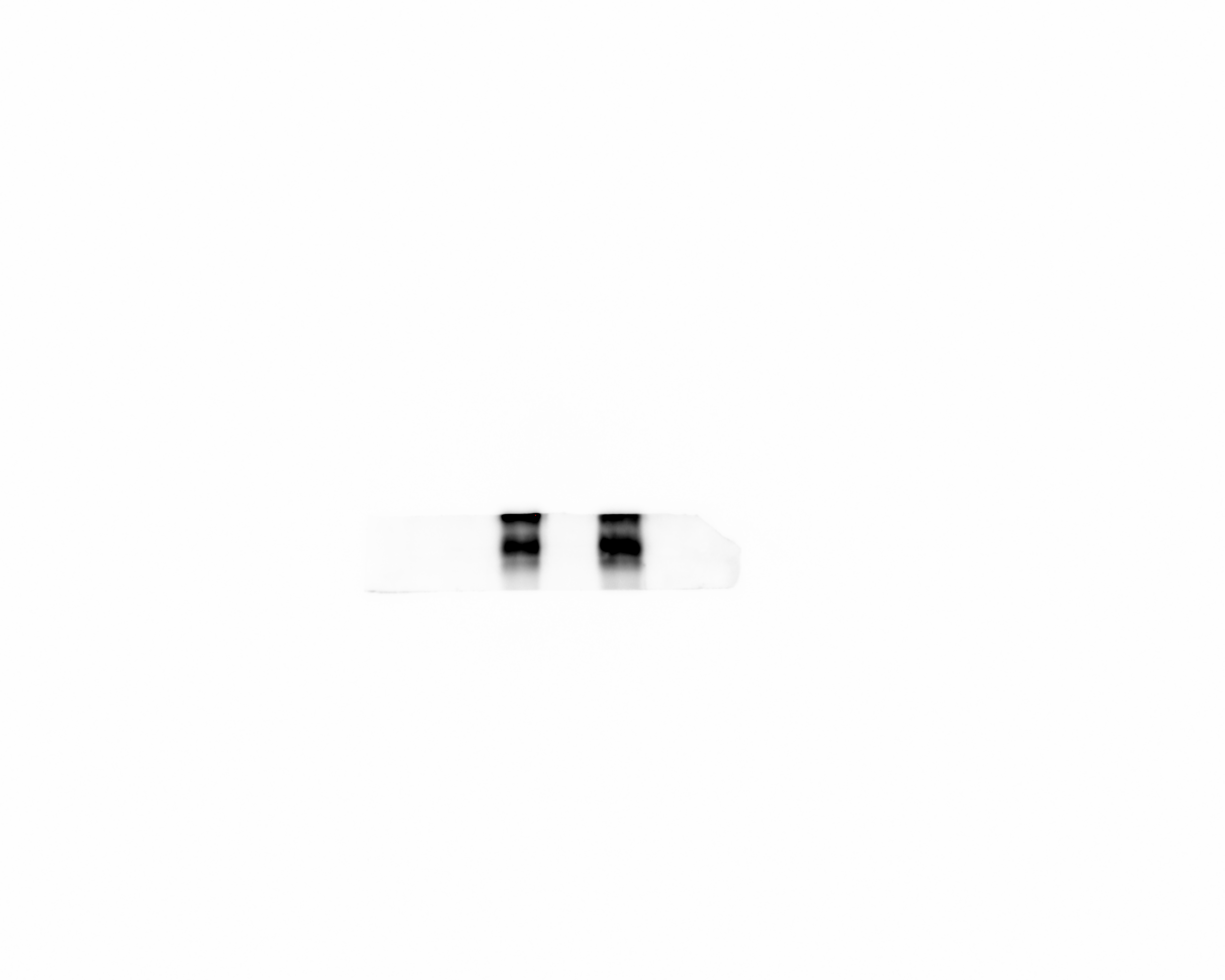

Supplement: Supplementary file 11 [file LSA-2019-00323_SdataF10.zip › Acharya_westernBlot_SourceData/2Ai_prashant 2018-12-06 04h39m09s p50ip panel1.tif]

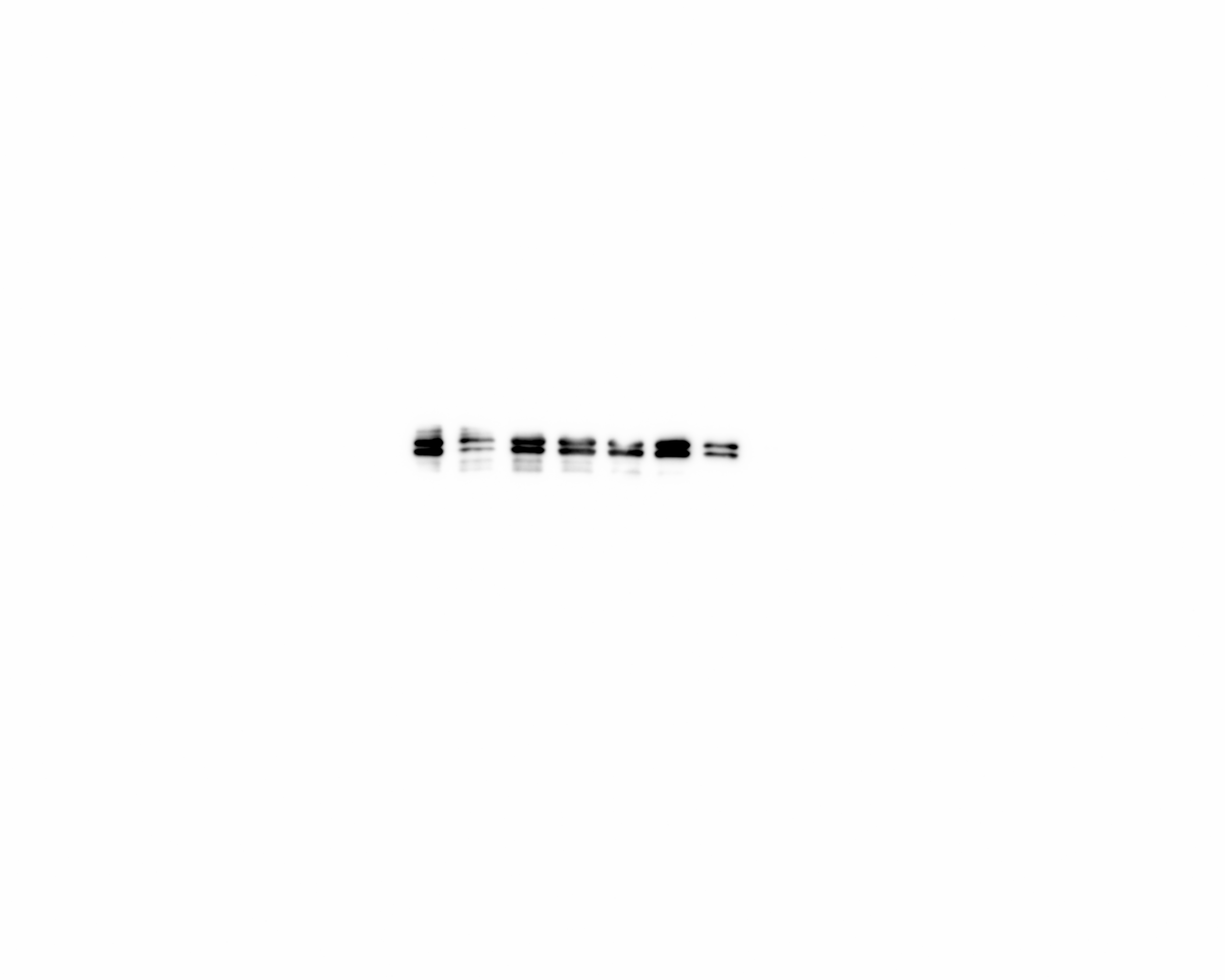

Supplement: Supplementary file 11 [file LSA-2019-00323_SdataF10.zip › Acharya_westernBlot_SourceData/6B_prashant 2018-12-26 03h33m31s p12 FOR flag p12.tif]

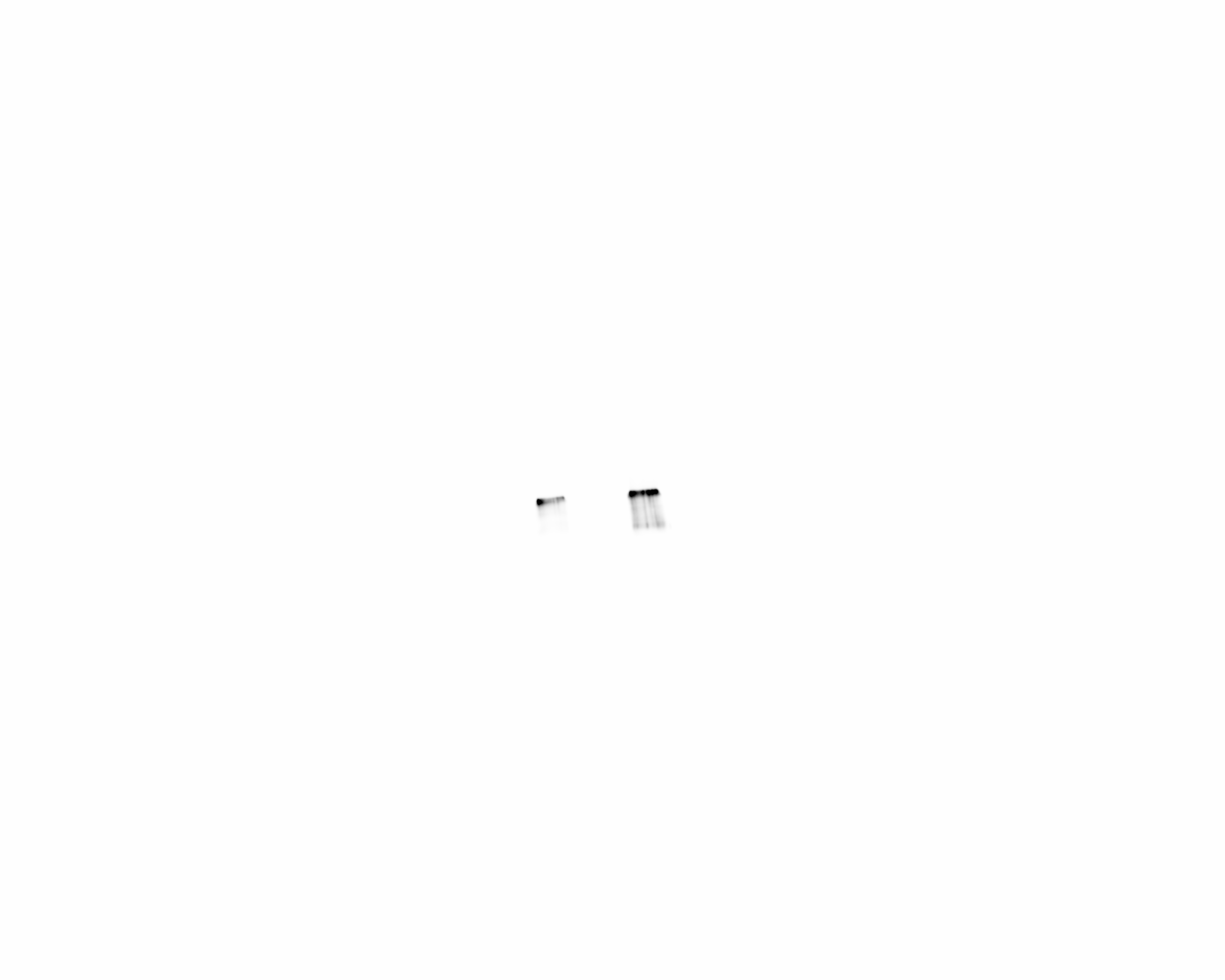

Supplement: Supplementary file 11 [file LSA-2019-00323_SdataF10.zip › Acharya_westernBlot_SourceData/2Ai_prashant 2018-12-04 04h53m53sp125 ip panel1.tif]
