# Supplementary material for: Human DNA polymerase delta is a pentameric holoenzyme with a dimeric p12 subunit
Source: Life Sci Alliance. 2019 Mar 18;2(2):e201900323. doi: 10.26508/lsa.201900323 (PMC6424025; doi:10.26508/lsa.201900323)

Raw data for Figure 7A .

PCNA Overlay assay

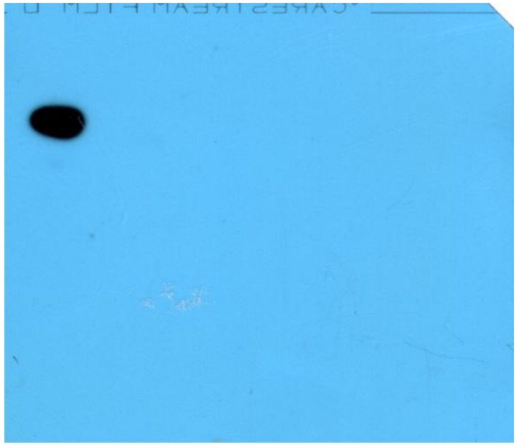

Raw data for Figure 7B .

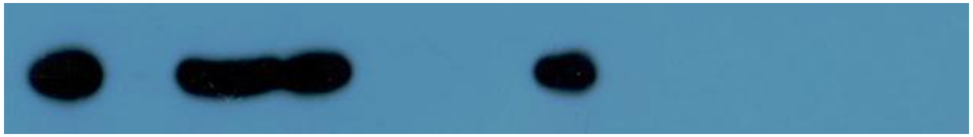

Raw plot for Figure 7C i(PCNA vs p12).

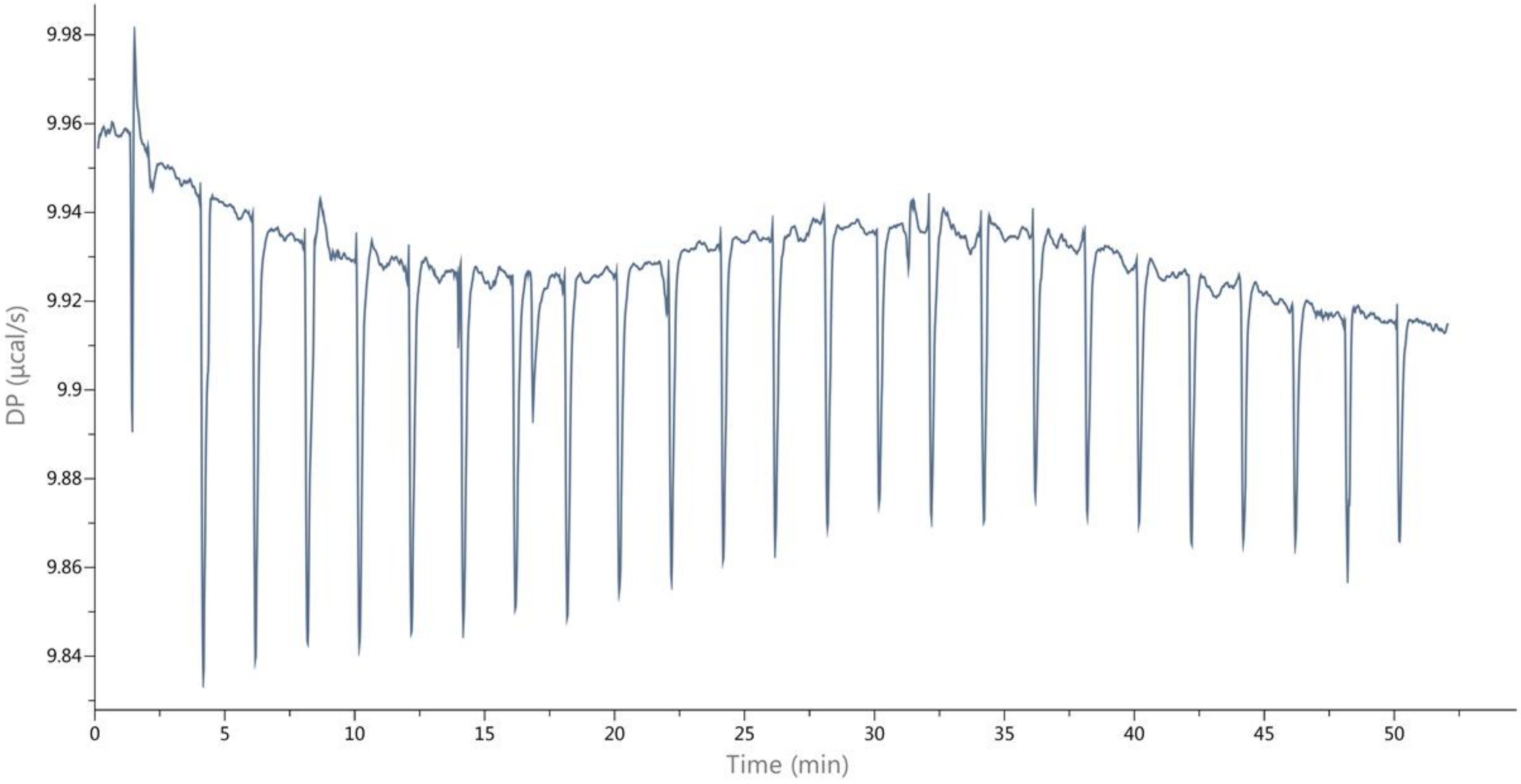

Raw plot for figure 7C ii (PCNA vs p12 RKR 3,4,5 AAA).

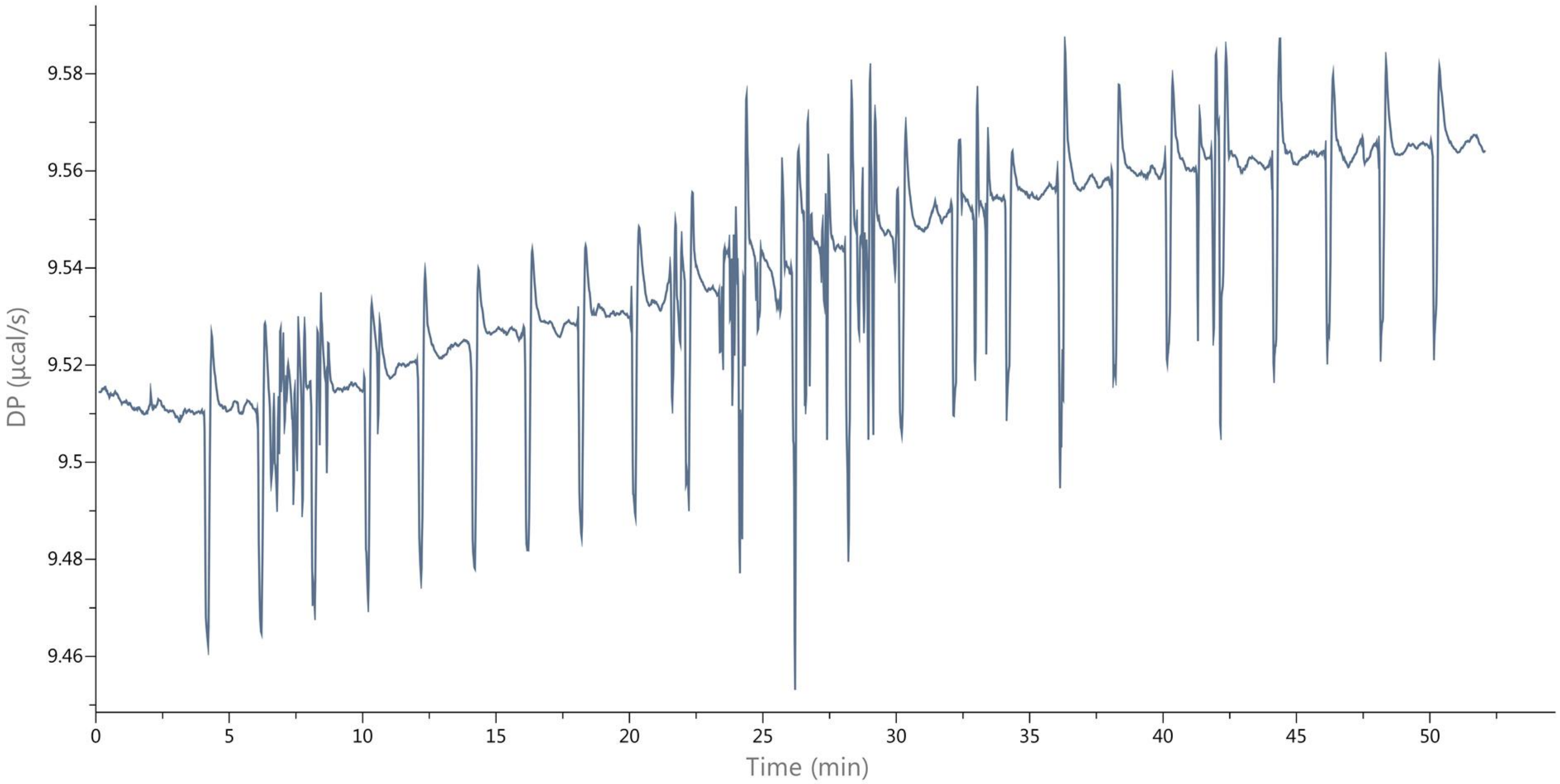

Raw plot for figure 7C iii(PCNA vs p12 LY 104, 105 AA).

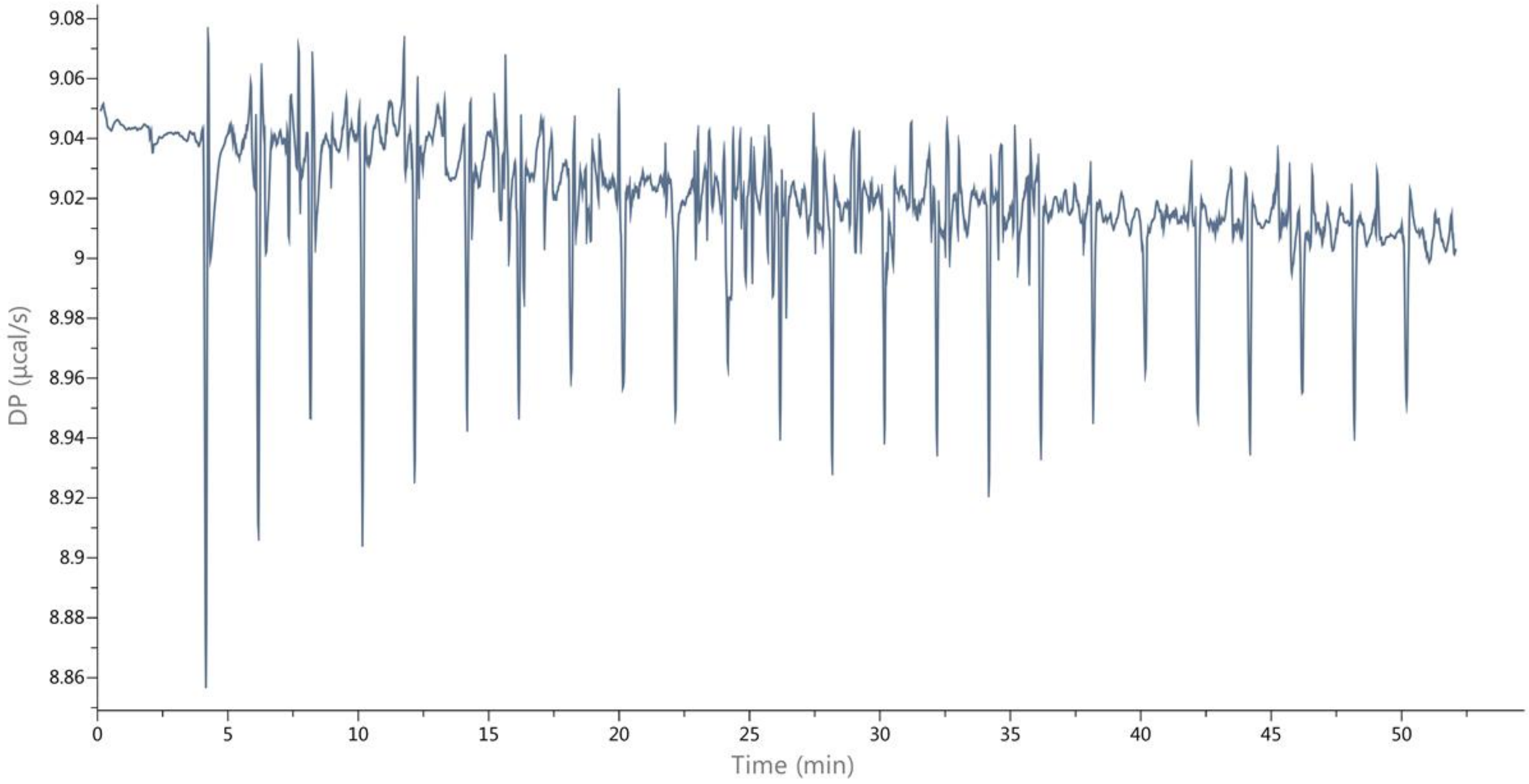

Supplement: Supplementary file 8 [file LSA-2019-00323_SdataF7.pdf]
