# Supplementary material for: Human DNA polymerase delta is a pentameric holoenzyme with a dimeric p12 subunit
Source: Life Sci Alliance. 2019 Mar 18;2(2):e201900323. doi: 10.26508/lsa.201900323 (PMC6424025; doi:10.26508/lsa.201900323)
Supplement: Supplementary file 12 [file LSA-2019-00323_TableS1.pdf]

Supplementary table- S1: List of primers used for various orfs amplification with underlined restriction enzymes.

Primer	Primer sequence (5'----3')	Name of the ORF
**NAP 239**	ccggggatccacatatgttcgaggcgcgcc	hPCNA
**NAP 240**	ccggggatccctaagatccttcttcatc	hPCNA
**NAP 251**	ccggggatccgtatgttcgaggcgcgcc	hPCNA
**NAP 300**	ctacacagctgtactcctgttctggagctccagcttgttcaacatc	hPCNA
**NAP 304**	gatgttgaacaagctggagctccagaacaggagtacagctgtgtag	hPCNA
**NAP 305**	ggccggatccctaagatccttcttcatcctcgaggcggcagccaag	hPCNA
**NAP 254**	ggccggatcctcaggggcccagccccagg	p50
**NAP 255**	ggcgaattcatgttttctgagcaggctgc	p50
**NAP 257**	ggccggatccttatttcctctggaagaagcc	p68
**NAP 258**	ggcgaattcatggcggaccagctttatctgg	p68
**NAP 260**	ccggggatccacatatgggccggaagcggctc	p12
**NAP 261**	ggccggatcctcataggggatagagatgcc	p12
**NAP 262**	ggcgaattcatgggccggaagcggctc	p12
**NAP 265**	ggccggatcctcataggggagcggcatgccagagactg	p12
**NAP 362**	ccggggatccgtatgggcgccgctgcactcatcactgattcc	p12
**NAP 373**	ggccggatccacatatgggcgccgctgcactcatcactg	p12
**NAP 252**	ggcgaattcatggatggcaagcggcgg	p125
**NAP 248**	ggccggatcctcaccaggcctcaggtccagggggtcc	p125
**NAP 361**	ggccggatccctagcctggaatggtttgaag	Cdm1
**NAP 451**	ccggggatccacatatgaagaagcgcac	Cdm1
**NAP 452**	ggccggatccacatatgactactcaagcgaaaaaatcaggg	Cdm1
**NAP 448**	cggcgaattctatgggccggaagcgg	p12
**NAP 450**	ccggggatccggtaggggatgatagagatg	p12
**NAP 444**	ccgggaattctatgctagaaaacaatgc	Polθ
**NAP 151**	ccgggtcgacggatccttacacatcaaagtcctttagctctcccc	Polθ
